# Exposure to hazardous air pollutants and risk of incident breast cancer in the nurses’ health study II

**DOI:** 10.1186/s12940-018-0372-3

**Published:** 2018-03-27

**Authors:** Jaime E. Hart, Kimberly A. Bertrand, Natalie DuPre, Peter James, Verónica M. Vieira, Trang VoPham, Maggie R. Mittleman, Rulla M. Tamimi, Francine Laden

**Affiliations:** 1 Channing Division of Network Medicine, Department of Medicine, Brigham and Women’s Hospital and Harvard Medical School, 401 Park Dr, Landmark Center, 3rd Floor West (BWH/HSPH), Boston, MA 02215 USA; 2 Exposure, Epidemiology, and Risk Program, Department of Environmental Health, Harvard T.H. Chan School of Public Health, Boston, MA USA; 3 Slone Epidemiology Center at Boston University, Boston, MA USA; 4 Department of Epidemiology, Harvard T.H. Chan School of Public Health, Boston, MA USA; 5 Department of Population Medicine, Harvard Medical School and Harvard Pilgrim Health Care Institute, Boston, MA USA; 6 Program in Public Health, University of California, Irvine, CA USA

**Keywords:** Air pollution, Hazardous air pollutants, Breast cancer

## Abstract

**Background:**

Findings from a recent prospective cohort study in California suggested increased risk of breast cancer associated with higher exposure to certain carcinogenic and estrogen-disrupting hazardous air pollutants (HAPs). However, to date, no nationwide studies have evaluated these possible associations. Our objective was to examine the impacts of mammary carcinogen and estrogen disrupting HAPs on risk of invasive breast cancer in a nationwide cohort.

**Methods:**

We assigned HAPs from the US Environmental Protection Agency’s 2002 National Air Toxics Assessment to 109,239 members of the nationwide, prospective Nurses’ Health Study II (NHSII). Risk of overall invasive, estrogen receptor (ER)-positive (ER+), and ER-negative (ER-) breast cancer with increasing quartiles of exposure were assessed in time-varying multivariable proportional hazards models, adjusted for traditional breast cancer risk factors.

**Results:**

A total of 3321 invasive cases occurred (2160 ER+, 558 ER-) during follow-up 1989–2011. Overall, there was no consistent pattern of elevated risk of the HAPs with risk of breast cancer. Suggestive elevations were only seen with increasing 1,2-dibromo-3-chloropropane exposures (multivariable adjusted HR of overall breast cancer = 1.12, 95% CI: 0.98–1.29; ER+ breast cancer HR = 1.09; 95% CI: 0.92, 1.30; ER- breast cancer HR = 1.14; 95% CI: 0.81, 1.61; each in the top exposure quartile compared to the lowest).

**Conclusions:**

Exposures to HAPs during adulthood were not consistently associated with an increased risk of overall or estrogen-receptor subtypes of invasive breast cancer in this nationwide cohort of women.

**Electronic supplementary material:**

The online version of this article (10.1186/s12940-018-0372-3) contains supplementary material, which is available to authorized users.

## Background

To date, known risk factors explain only half of all breast cancer diagnoses, demonstrating a need to identify additional targets for prevention [1–3]. Geographic differences in breast cancer rates, even after adjustment for differences in distributions of risk factors, have led to an increasing interest in the environment as a potential source of population-level risk factors for breast cancer [4–12]. Findings of higher breast cancer risk in urban areas have given focus to exposures in urban settings such as air pollution. Ambient air pollution was recently classified as a human carcinogen, mainly based on the evidence for lung cancer [13]. A growing body of research has examined factors such as air pollution and traffic exposures as risk factors for breast cancer, however; to date, the findings have been mixed [14–27].

Two recent analyses based in the California Teachers Study (CTS) cohort (a study of mostly postmenopausal women, of whom 26% were nulliparous) have focused on Hazardous Air Pollutants (HAPs) as another potential group of environmental risk factors for breast cancer [26, 27]. HAPs are chemicals that are known to cause cancer or have other adverse health effects, and are regulated and monitored by the US Environmental Protection Agency (EPA). Using information available from EPA, Garcia et al. examined the association of HAPs that had been previously identified from toxicological studies as mammary carcinogens and risk of invasive breast cancer [26]. Of the 24 HAPs examined, acrylamide, carbon tetrachloride, chloroprene, 4,4′-methylene bis(2-chloroaniline), propylene oxide, and vinyl chloride were associated with an increased risk of breast cancer overall. Many of the HAPs displayed non-linear dose-responses, and the tests for trend were only statistically significant for acrylamide, carbon tetrachloride, chloroprene, 4,4′-methylene bis(2-chloroaniline), and vinyl chloride. Acrylamide, benzidine, carbon tetrachloride, ethylidene dichloride, and vinyl chloride were associated with increased risks of estrogen (ER) and progesterone (PR) receptor-positive tumors (ER+/PR+), and benzene was associated with an increased risk of receptor-negative (ER-/PR-) tumors.

In a related analysis, Liu et al. examined associations with HAPs that had been previously identified as estrogen disruptors and risk of breast cancer in the CTS [27]. There was little evidence of elevated risks with increasing exposure in the full cohort, but there were suggestions of elevated risks of hormone receptor-negative cancers with exposures to cadmium. The results of these two previous studies suggest that HAPs may be a previously unidentified risk factor for breast cancer, and that these associations should be examined in other cohorts with a larger geographic scale and among women of different ages. Therefore, the purpose of our study was to assess the impacts of HAPs among a nationwide cohort of mostly premenopausal women, the Nurses’ Health Study II (NHSII).

## Methods

### Study population

The NHS II is a prospective cohort established in 1989 when 116,430 female nurses aged 25–42 years completed a baseline questionnaire. At enrollment, the women resided in 14 states (California, Connecticut, Indiana, Iowa, Kentucky, Massachusetts, Michigan, Missouri, New York, North Carolina, Ohio, Pennsylvania, South Carolina, and Texas). However, as of the mid-1990’s, members of the cohort resided in all 50 states and the District of Columbia. Biennial follow-up questionnaires are mailed with response rates over 90% and collect information on incidence of disease and on a variety of lifestyle characteristics. Women were excluded from the current study if they had been diagnosed with cancer (other than non-melanoma skin cancer) before returning the baseline questionnaire. Women were censored during follow-up if they were diagnosed with a cancer other than breast or non-melanoma skin cancer. Participants were excluded if they lived outside of the contiguous US, or if they lived in a Census tract with no information available on exposure. After these exclusions, there were a total of 109,239 women available for analysis. The study was approved by the Institutional Review Board (IRB) of Brigham and Women’s Hospital and participants provided implied consent through the return of questionnaires.

### Outcome assessment

Participants self-report newly diagnosed invasive breast cancers on the biennial questionnaires. Deaths are ascertained from family members, the US Postal Service (in response to cohort mailings), or through annual searches of the National Death Index. Medical records were reviewed by study staff without knowledge of exposure status. In this cohort, pathology reports have been shown to confirm 99% of self-reported diagnoses of breast cancer. Therefore, we included self-reported cases where medical record information was not available in our analyses. Tissue block collection and tissue microarray (TMA) construction were described in detail previously [28] and immunohistochemical (IHC) analysis was performed according to a standard protocol [29]. Cases were considered estrogen receptor ER+ if any tissue core showed any nuclear staining for ER. Cases with complete absence of staining for ER were considered ER-. For cases where ER status was not available from IHC on the TMAs, ER status was based on information available in the pathology report or medical record. Each case was also classified as premenopausal or postmenopausal based on information provided by the participant on the biennial follow-up questionnaire at the time of the case report.

### Exposure assessment

As part of the questionnaire mailing process, residential address histories are available for each participant from 1989 forward. Each address has been geocoded to obtain latitude and longitude, as well as Census geographies for each decennial Census. Ambient concentrations of HAPs identified as potential mammary carcinogens or estrogen disruptors were assigned to each residential address using information from the US Environmental Protection Agency’s National Air Toxics Assessment (NATA) [30]. This program was developed to identify and prioritize air toxics based on their population-level health risks. NATA assessments have been conducted in 1996 (33 HAPs), 1999 (177 HAPs), 2002 (181 HAPs), and 2005 (178 HAPs) to provide estimates of HAPs for all Census tracts in the US. Details of the prediction process are discussed in detail elsewhere. [31] Briefly, to obtain predictions, a national inventory of outdoor sources of emissions is first assembled. Next, dispersion modeling is used to combine the emissions information with meteorological data to determine average annual concentrations of each HAP for all Census tracts. EPA cautions against comparing concentrations across NATA years due to differences in methodology, therefore, we chose to use the 2002 NATA estimates to approximate the predictions used in the CTS. The values from the 2002 NATA were assigned to all addresses in each residential address history. Therefore, only women who moved would have different values for each HAP throughout follow-up. In sensitivity analyses to determine if the choice of NATA influenced results, we also conducted analyses using the 1996 NATA.

We used an approach parallel to that used in the CTS to select HAPs of interest [26, 27]. These included 37 compounds that were assessed in the 2002 NATA that were identified in toxicological studies as mammary carcinogens and 9 potential estrogen disrupting compounds identified either from the Institute of Environment and Health of the University of Leicester or the Endocrine Disruptor Exchange Inc., available in the 2002 NATA [32–34]. Twelve of these compounds were also available in the 1996 NATA. We assessed the distributions of each compound across the full residential address histories of our participants and excluded HAPs for which there was not sufficient variability to create quartiles of exposure or if zeros comprised more than 25% of the distribution. Twenty-three mammary carcinogens were ultimately selected [1,2-dibromo-3-chloropropane, 1,3-butadiene, 2,4-dinitrotoluene, 2,4-toluene diisocyanate, 2-chloroacetophenone, acrylonitrile, benzene (including benzene from gasoline), benzidine, chloroprene, diesel engine emissions, ethylene dibromide (dibromoethane), ethylene dichloride (1,2-dichloroethane), ethylene oxide, ethylidene dichloride (1,1-dichloroethane), hydrazine, methylene chloride (dichloromethane), nitrobenzene, o-toluidine, propylene dichloride (1,2-dichloropropane), propylene oxide, styrene, vinyl chloride, and vinylidene chloride (1,1-dichloroethylene)]. We additionally included 1,4-dioxane and carbon tetrachloride for comparison to the CTS results. The estrogen disrupting chemicals selected for analysis included 4-nitrophenol, inorganic arsenic compounds, biphenyl, bis(2-ethylhexyl)phthalate (DEHP), dibutulphthalate, diesel engine emissions, dimethyl formamide, selenium compounds, and styrene.

### Potential confounders

Information on potential risk factors and effect modifiers is obtained on each biennial follow-up questionnaire (or every other questionnaire for physical activity and dietary factors). We considered a number of risk factors for invasive breast cancer, or predictors of exposure including: race, height, body mass index (BMI) at age 18 and difference between current BMI and BMI at age 18, alcohol consumption at ages 15 and 18, smoking status, diet quality (including alcohol consumption) as assessed by the Alternative Healthy Eating Index, [35] physical activity, history of aspiration or biopsy confirmed benign breast disease, family history of breast cancer, age at menarche, parity and age at first birth, use of oral contraceptives, menopausal status and postmenopausal hormone use, screening mammogram within the past 2 years, history of rotating shift work (ever vs never), individual-level socioeconomic status (SES) (marital status, household income, and if the participant lived alone or with others), area-level SES (Census tract level median income and median home value), and Census region of residence as potential confounders. Multivariable models contained all a priori confounders. To determine the importance of specific potential confounders (or sets of potential confounders), each was added to models adjusted for age and calendar period separately to determine its impact on the effect estimates.

### Statistical analyses

Person-months of follow-up were calculated from June 1989 through the end of follow-up (May 2011), incidence of invasive breast cancer, incidence of other cancer (including in situ breast cancers), death, or loss to follow-up, whichever occurred first. Time-varying Cox proportional hazards models were used to assess the association of incident invasive breast cancer with quartiles of each compound, and hazard ratios (HRs) and 95% confidence intervals (CIs) were calculated comparing each quartile to the lowest. Quartiles of exposure were used after cubic regression splines indicated that several dose-responses statistically significantly deviated from linearity. *P*-values for trend were obtained from models using the median value for each quartile of exposure. All models were stratified by current age in months and questionnaire cycle to control for age and temporal effects. We performed sensitivity analyses examining the associations in subgroups of interest including models restricted to ER+ or ER-, analyses among women who were currently pre-menopausal, and models stratified by smoking status (ever vs never).

As noted above, we conducted sensitivity analyses using estimates from the 1996 NATA to determine if the choice of predictions affected our results. We also assessed Pearson and Spearman correlations between the 1996 and 2002 predictions of each HAP.

## Results

Age-standardized characteristics of the cohort throughout the full period of follow-up are presented in Table 1. The average age of the women was 44.4 ± 7.8 years. They were predominantly white (95%), 65% never smoked, and 16% had a previous history of benign breast disease. Eighteen percent were nulliparous, 24% were less than 12 years at menarche, and 72% were premenopausal. Most of the women lived in the Northeast (31%) or Midwest (34%). Distributions of each of the HAPs are shown in Additional file 1: Table S1. The average concentrations of the compounds varied, ranging from 1.73 × 10^− 7^ μg/m^3^ for benzidine to 1.34 μg/m^3^ for benzene, with the majority of compounds having their full range of concentrations below 1 μg/m^3^.

**Table 1:** Selected age-standardized characteristics of the 109,239 members of the Nurses’ Health Study II throughout follow-up (1989–2011)

Characteristic	Mean (SD) or %
Age (years)^a^	44.39 (7.81)
Physical activity (MET-hrs/week)	19.58 (27.81)
Body mass index (BMI) at age 18 (kg/m^2^)	21.22 (3.17)
Difference between current BMI and BMI at age 18	5.03 (5.15)
White	95
History of biopsy confirmed benign breast disease	16
Family history of breast cancer	11
Screening mammogram in past 2 years	50
Age at menarche (years)	
< 12	24
12	30
13	27
14+	18
Parity	
Nulliparous	18
Parous, by number of children	
1–2	47
3–8	24
Height (inches)	
50 < − < 62	9
62- < 65	37
65- < 68	38
68+	16
Oral contraceptive use	
Never	12
Past	73
Current	7
Menopausal status and hormone use	
Premenopausal	72
Postmenopausal never users	4
Postmenopausal past users	9
Postmenopausal current users	8
Smoking status	
Never	65
Past	24
Current	9
Rotating Shift Work	
Never	32
1–9 years	62
≥ 10 years	6
Individual level socioeconomic status	
Married	56
Not Married	44
Live alone	7
Live with others	93
Personal income in 2001	
less than 100,000	40
100–149,000	14
150,000+	8
Not reported	38
Census tract level socioeconomic status	
Median home value	160,490 (121,757)
Median income	62,781 (23,818)
Region of residence	
Northeast	31
Midwest	34
West	15
South	20

^a^Value is not age adjusted

During 2,203,192 years of follow-up, a total of 3321 invasive cases occurred (2160 ER+, 558 ER-, and 603 with unknown ER status). Overall, there was little consistent evidence of adverse effects of increasing exposure to any of the mammary carcinogens with increasing risk of invasive breast cancers (Additional file 2). Elevations in risk with increasing exposure were observed for 1,2-dibromo-3-chloropropane (top quartile multivariable adjusted HR = 1.12, 95% CI: 0.98–1.29, p-for trend = 0.004). Diesel exhaust and hydrazine exhibited elevations in risk that did not appear to increase with increasing exposures. Statistically significant reductions in risk were observed with increasing exposures to 2-chloroacetophenone (top quartile HR = 0.84, 95% CI: 0.75, 0.94, p-for-trend = 0.001 and chloroprene (top quartile HR = 0.89, 95% CI: 0.80, 0.99, p-for-trend = 0.01), and exposures to ethylene dibromide and o-toluidine exhibited reductions in risk that were not dose-dependent.

With the exception of 1,2-dibromo-3-chloropropane (increased risk), hydrazine (decreased risk), and 2-chloroacetophenone (decreased risk), none of the examined HAPs had a statistically significant dose-response with risk of ER+ cancers. Elevations in risk were observed with some exposures (1,3,-butadiene, 2,4-dinitrotoluene, 2,4,-toluene diisocyanate, benzene, carbon tetrachloride, diesel exhaust, nitrobenzene, propylene oxide, and vinylidene chloride) and reductions in risk with others (1,4-dioxane, 2-chloroacetophenone, acrylonitrile, ethylene dibromide, and propylene dichloride) (Additional file 2). There were no statistically significant dose-responses observed between any of the HAPs with risk of ER- cancers. Suggestive elevations in risk were observed with exposures to 1,2-dibromo-3-chloropropane, ethylene oxide, hydrazine, and vinyl chloride. Suggestive reductions in risk were observed with increasing exposures to acrylonitrile, benzene, benzidine, chloroprene, and o-toluidine.

There was also little consistent evidence of adverse effects of increasing exposure to the estrogen disruptor HAPs with increasing risk of invasive breast cancers (Table 2). Diesel exhaust and 4-nitrophenol exhibited positive dose-responses (top quintile diesel HR = 1.10; 95% CI: 0.99, 1.22; 4-nitrophenol HR = 1.07 (95% CI:0.96, 1.19) with overall risk of invasive breast cancer, but neither p-for-trend reached statistical significance. Suggestive elevations were also observed with exposures to dibutulphthalate and dimethyl formamide. Similar patterns were observed for risk of ER+ cancers, while risk of ER- cancers was suggestively associated with increases in exposure to bis(2-ethylhexyl)phthalate and decreases in exposures to dimethyl formamide.

**Table 2:** Multivariable adjusted associations of increasing quartiles of each estrogen disruptor HAP exposure on risk of incident invasive overall, estrogen-receptor positive (ER+) or estrogen-receptor negative (ER-) breast cancer 1989–2011 among 109,239 members of the Nurses’ Health Study II cohort

		Quartile 1		Quartile 2		Quartile 3		Quartile 4		
Hazardous air pollutant	Outcome	Cases	HR (95% CI)	Cases	HR (95% CI)	Cases	HR (95% CI)	Cases	HR (95% CI)	*P*-value for trend
Diesel engine emissions^a^	Overall	795	Ref	828	1.03 (0.93, 1.14)	828	1.02 (0.92, 1.13)	870	1.10 (0.99, 1.22)	0.096
	ER+	518	Ref	530	1.02 (0.90, 1.15)	540	1.03 (0.91, 1.17)	572	1.11 (0.97, 1.27)	0.099
	ER-	139	Ref	152	1.07 (0.84, 1.35)	146	1.02 (0.80, 1.32)	121	0.91 (0.70, 1.19)	0.401
Arsenic compounds (Inorganic)	Overall	812	Ref	855	0.99 (0.90, 1.10)	846	0.97 (0.88, 1.07)	808	0.96 (0.86, 1.06)	0.321
	ER+	529	Ref	533	0.95 (0.84, 1.08)	560	0.99 (0.87, 1.13)	538	0.97 (0.86, 1.10)	0.837
	ER-	146	Ref	152	1.01 (0.80, 1.28)	128	0.88 (0.68, 1.13)	132	0.91 (0.71, 1.16)	0.319
Biphenyl	Overall	801	Ref	859	1.06 (0.96, 1.17)	857	1.07 (0.97, 1.18)	804	0.99 (0.89, 1.09)	0.293
	ER+	522	Ref	567	1.09 (0.96, 1.23)	541	1.05 (0.93, 1.19)	530	1.01 (0.89, 1.15)	0.640
	ER-	140	Ref	137	0.98 (0.77, 1.25)	161	1.18 (0.94, 1.50)	120	0.93 (0.71, 1.21)	0.418
Bis(2-Ethylhexyl)Phthalate (Dehp)	Overall	833	Ref	809	0.99 (0.90, 1.10)	867	1.06 (0.96, 1.17)	812	1.01 (0.92, 1.12)	0.756
	ER+	565	Ref	516	0.96 (0.84, 1.09)	559	1.03 (0.91, 1.16)	520	0.98 (0.87, 1.11)	0.897
	ER-	131	Ref	145	1.09 (0.85, 1.39)	137	1.06 (0.82, 1.36)	145	1.22 (0.96, 1.56)	0.110
Dibutulphthalate	Overall	797	Ref	790	0.96 (0.87, 1.06)	902	1.13 (1.02, 1.25)^*^	832	1.06 (0.96, 1.17)	0.414
	ER+	525	Ref	530	0.99 (0.87, 1.12)	571	1.10 (0.97, 1.24)	534	1.05 (0.93, 1.19)	0.600
	ER-	143	Ref	135	0.95 (0.74, 1.21)	146	1.12 (0.87, 1.43)	134	0.98 (0.76, 1.24)	0.716
Dimethyl formamide	Overall	814	Ref	813	1.01 (0.91, 1.12)	867	1.08 (0.97, 1.19)	827	1.08 (0.97, 1.20)	0.105
	ER+	531	Ref	521	1.00 (0.88, 1.13)	579	1.13 (0.99, 1.28)	529	1.09 (0.95, 1.24)	0.145
	ER-	149	Ref	138	0.94 (0.74, 1.19)	144	0.97 (0.76, 1.24)	127	0.93 (0.72, 1.20)	0.683
4-Nitrophenol	Overall	798	Ref	836	1.01 (0.91, 1.12)	832	1.01 (0.91, 1.12)	855	1.07 (0.96, 1.19)	0.179
	ER+	510	Ref	563	1.07 (0.95, 1.22)	544	1.04 (0.92, 1.19)	543	1.06 (0.92, 1.21)	0.655
	ER-	144	Ref	130	0.90 (0.70, 1.15)	150	1.07 (0.84, 1.36)	134	1.02 (0.79, 1.32)	0.610
Selenium compounds	Overall	802	Ref	837	1.04 (0.94, 1.14)	882	1.06 (0.96, 1.17)	800	0.96 (0.86, 1.07)	0.283
	ER+	516	Ref	546	1.06 (0.94, 1.20)	578	1.08 (0.95, 1.22)	520	0.95 (0.83, 1.09)	0.230
	ER-	150	Ref	141	0.97 (0.77, 1.22)	146	1.00 (0.79, 1.27)	121	0.84 (0.64, 1.11)	0.222
Styrene^a^	Overall	1050	Ref	632	0.97 (0.88, 1.07)	753	0.94 (0.85, 1.03)	886	0.97 (0.89, 1.06)	0.676
	ER+	681	Ref	400	0.96 (0.85, 1.09)	495	0.97 (0.86, 1.09)	584	1.01 (0.90, 1.13)	0.744
	ER-	176	Ref	113	1.05 (0.83, 1.34)	131	0.99 (0.78, 1.24)	138	0.92 (0.73, 1.15)	0.341

*Note:* All models adjusted for age, calendar period, race, family history of breast cancer, history of aspiration or biopsy confirmed benign breast disease, age at menarche, parity and age at first birth, menopausal status and postmenopausal hormone use, oral contraception use, recent mammogram, height, BMI at age 18, difference between current BMI and BMI at age 18, smoking status, physical activity, overall diet quality (including alcohol consumption), alcohol consumption at age 15 and age 18, shift work, individual-level SES (marital status, living arrangements, household income), area-level SES (Census tract median home value and median income), and Census region of residence

^a^Diesel exhaust and Styrene are both potential estrogen disruptors and mammary carcinogens

^*^indicates *p*-values< 0.05

Measures of area-level SES and parity and age at first birth appeared to be the only confounders in most models (see examples in Additional file 1: Figure S1). Patterns of association with each of the HAPs were similar, although somewhat further from the null, in models restricted to women who were premenopausal (Additional file 1: Table S2). Results were also similar in models restricted to never smokers (Additional file 1: Table S3) or ever smokers (Additional file 1: Table S4).

The Pearson correlations between exposures based on the 1996 and 2002 NATA assessments, although statistically significant, tended to be small to modest (Additional file 1: Table S5). However, based on the Spearman correlations, the rank ordering of Census tracts did appear to be relatively consistent between the two time points. Models using the values from 1996 assessment had generally similar patterns as those using the 2002 values (Additional file 1: Table S6).

## Discussion

In this nationwide prospective cohort study of women, we observed limited evidence of adverse effects of HAPs with increased risks of invasive, ER+, and ER- breast cancer. Suggestive increases in risk were only consistently observed with increasing exposures to 1,2-dibromo-3-chloropropane. Overall, our findings were not similar to those observed in the mostly post-menopausal CTS cohort [26, 27].

Our findings of consistent elevations of risk with increasing exposures to 1,2-dibromo-3-chloropropane could not be compared to findings from the CTS, as 1,2-dibromo-3-chloropropane did not have enough exposure variability to be included in their analyses. 1,2-dibromo-3-chloropropane is a pesticide previously used as a soil fumigant to eliminate nematodes [1]. It was banned in 1977 for all applications except application to pineapples, and was fully banned in 1985, but has been detected in samples of ambient air. Inhalation is not surmised to be the dominant pathway of exposure, as current exposures are thought to be dominated by ingestion of contaminated drinking water and food grown in soils historically treated with 1,2-dibromo-3-chloropropane. In toxicological studies, 1,2-dibromo-3-chloropropane was associated with mammary tumor formation in rats, primarily in studies where 1,2-dibromo-3-chloropropane was administered via ingestion, not inhalation [1, 32].

The CTS observed elevations in overall invasive breast cancer risk with increasing exposures to several mammary carcinogens: acrylamide, carbon tetrachloride, chloroprene, 4,4′-methylene bis(2-chloroaniline), and vinyl chloride (all p-for-trends< 0.05) [26]. We did not observe similar patterns, except with diesel exhaust emissions (discussed below). In our cohort, there was not enough variability in exposures to acrylamide or 4,4′-methylene bis(2-chloroaniline) to assess their association with breast cancer risk. In the CTS, comparing the fifth quintile of exposure to carbon tetrachloride to the first, the HR from basic models (adjusted only for age and race) was 1.08 (95% CI: 1.00, 1.18), our multivariable adjusted HR was 1.02 (95% CI: 0.64, 1.63), comparing women with non-zero values to those with zeros. These are in line with suggestive elevations in breast cancer risk with exposure to carbon tetrachloride reported in a case-control study of mortality records, where a job exposure matrix was used to determine probability and level of exposure, [36] in a small study of aircraft maintenance workers exposed occupationally, [37] and in an ecological study of comparing rates between counties with an without documented releases. [38] For chloroprene, the CTS observed elevations in risk, although only 3 groups of exposure were possible; we observed a statistically significant decrease in risk with increasing exposure across quartiles of exposure. Lastly, exposures to vinyl chloride were associated with increased risks across the 4 top quintiles of exposure in CTS in some models, and with an inverse-U shape in others, while we only observed non-statistically significant elevations in the 3rd quartile. No other epidemiologic studies have examined exposures to vinyl chloride, although there is a large body of evidence from animal models suggesting that vinyl chloride can induce mammary cancers [6].

The CTS investigators reported elevations in risk of ER+/PR+ breast cancers and exposures to acrylamide, benzidine, carbon tetrachloride, ethylidene dichloride, and vinyl choloride and for risk of ER-/PR- breast cancers and benzene. With the exception of a suggested elevated risk in risk of ER+ breast cancer comparing women with non-zero exposures to carbon tetrachloride to women with a level of zero (HR = 1.06; 95% CI: 0.60, 1.88), we did not observe similar findings in the NHSII.

We observed a suggestive increased risk of invasive breast cancer with exposures to two estrogen disruptors, diesel engine emissions (also considered as a mammary carcinogen in our study: top quartile HR = 1.10; 95% CI: 0.99, 1.22) and 4-nitrophenol (top quartile HR = 1.07; 95% CI: 0.96, 1.19). The CTS investigators reported that they did not observe an association between any of the examined estrogen disruptors and risk of breast cancer, however their multivariable adjusted HR for the top quintiles of diesel engine emissions (HR = 1.04; 95% CI: 0.95, 1.13) and 4-nitrophenol (HR = 1.07; 95% CI: 0.98, 1.17) were also elevated [27]. Both diesel exhaust particulate and nitrophenols from diesel exhaust have been shown to have hormonal and cancer-promoting effects [39–42]. Exposures to diesel exhaust, or other measures of traffic exposure, were associated with an increased risk of breast cancer in several other studies [43–50]. Therefore, even the small risks reported in our study could lead to a major public health burden, given the sizable portion of the population exposed to diesel exhaust.

It is important to note that there are a number of differences between the CTS and NHSII cohorts. The cohorts are quite different spatially (California vs. the full US) and the women in the CTS were less likely to be white (85% in CTS compared to 95% in NHSII) and were much more likely to be postmenopausal [27]. Women in the CTS were more likely to be nulliparous (26% compared to 18% in the NHSII), but had similar proportions of never smokers and distributions of age at menarche. These differences may partially explain the differences in our findings, especially if the impact of the examined HAPs is more important for risk of postmenopausal breast cancers.

Our study has a number of limitations. The use of the US EPA HAPs data as our source of exposure may have introduced exposure misclassification in a number of ways. Although we used updated address information, the HAPs estimates were chosen from a single year, 2002. This decision was made to increase comparability with the CTS analyses, and based recommendations from EPA not to use multiple NATA assessments due to differences in the methodology. However, this means that we are unable to assess the impacts of concentration changes over time. Another source of exposure error is the use of a Census tract level measure as an estimate of personal exposures for each participant. The use of an area-level proxy likely leads to substantial nondifferential exposure misclassification, as it does not take into account differences within a Census tract, differences in the amount of time each participant spends in the Census tract where she lives, and differences in the amount of time each woman would be exposed to these specific ambient concentrations. We are also unable to account for exposures to any of these HAPs from indoor sources, or from other routes of exposure. Importantly, for this population, who may have been exposed to some of these chemicals as nurses, we are unable to account for exposures to these chemicals at work, as NHSII does not have information on workplace chemical exposures. The potential for substantial exposure error may explain the overall lack of associations in our study.

Another limitation of our analysis is the inability to examine exposures early in the life of our participants. Studies have demonstrated that exposures in early childhood and adolescence, and before first birth may be more strongly associated with risk of breast cancer than exposures later on in life [6, 51]. However, due to the design of the NHSII cohort and the availability of HAPs data, we were unable to examine exposures during key windows of potential susceptibility. The average age of our cohort throughout follow-up was 44.4 years old, therefore we are only able to assess the impacts of these exposures during adulthood. And, as a number of the HAPs of interest in this study were banned at the time of the 2002 NATA assessment, but in use during key windows of life for these participants, it would not be appropriate to assume that these exposures could be assigned to addresses during high school or other key time points.

Lastly, although this is the first nationwide study to be able to examine the impacts of mammary carcinogens and estrogen disruptor HAPs available from the 2002 NATA, our findings may not be generalizable to all populations of women in the US. Participants in the NHSII do not tend to live in neighborhoods with low SES, which may be disproportionally impacted by emissions of these HAPs from industrial sources. If there are interactions between measures of socioeconomic disadvantage and HAPs on risk of breast cancer, then our results would not be generalizable to groups with lower SES.

Our study also has a number of strengths. Participants of the NHSII live in all of the 48 contiguous US and the District of Columbia, allowing us to examine the impacts of these HAPs across the US. The restriction of our cohort to a single occupational group and the availability of time-varying information on a wide variety of personal- and area-level potential confounders allowed us to tightly control for potential confounding. Lastly, the large size of the cohort, combined with over 20 years of follow-up, allowed us sufficient power to detect small differences in exposure if they had been present.

## Conclusions

In this nationwide cohort of women, exposures to HAPs determined to be mammary carcinogens or estrogen disruptors during adulthood were generally not associated with an increased risk of overall or hormone-receptor subtype specific risk of breast cancer. Additional studies are needed to determine if these exposures are associated with risk of breast cancer, especially during key windows of potential susceptibility.

## Additional files

Additional file 1: Supplemental tables and figures. (DOCX 284 kb)

Additional file 2: Multivariable adjusted associations of increasing quartiles of each mammary carcinogen HAP exposure on risk of incident invasive overall, estrogen-receptor positive (ER+) or estrogen-receptor negative (ER-) breast cancer 1989–2011 among 109,239 members of the Nurses’ Health Study II cohort (DOCX 51 kb)

## Abbreviations

BMI: Body mass index
CI: Confidence interval
CTS: California Teachers’ Study
DEHP: Bis(2-ethylhexyl)phthalate
EPA: US Environmental Protection Agency
ER: Estrogen receptor
HAPs: Hazardous air pollutants
HR: Hazard ratio
NATA: National air toxics assessment
NHSII: Nurses’ Health Study II
SES: Socioeconomic status
US: United States of America
