# Supplementary material for: Exposure to hazardous air pollutants and risk of incident breast cancer in the nurses’ health study II
Source: Environ Health. 2018 Mar 27;17:28. doi: 10.1186/s12940-018-0372-3 (PMC5870204; doi:10.1186/s12940-018-0372-3)
Supplement: Supplementary file 1 — Supplemental tables and figures. (DOCX 284 kb) [file 12940_2018_372_MOESM1_ESM.docx]

**Additional file**

**Exposure to Hazardous Air Pollutants and Risk of Incident Breast Cancer in the Nurses’ Health Study II**

Jaime E. Hart^1,2^, Kimberly A. Bertrand^3^, Natalie DuPre^4^, Peter James^5^, **Verónica M. Vieira^6^**, Trang VoPham^1,4^, Maggie R. Mittleman,^1^ Rulla M. Tamimi^1,4*^, Francine Laden^1,2,4*^

^1^ Channing Division of Network Medicine, Department of Medicine, Brigham and Women’s Hospital and Harvard Medical School, Boston, MA, USA

^2^ Exposure, Epidemiology, and Risk Program, Department of Environmental Health, Harvard T.H. Chan School of Public Health, Boston, MA, USA

^3^ Slone Epidemiology Center at Boston University, Boston, MA, USA

^4^ Department of Epidemiology, Harvard T.H. Chan School of Public Health, Boston, MA, USA

^5^ Department of Population Medicine, Harvard Medical School and Harvard Pilgrim Health Care Institute, Boston, Massachusetts, USA

^6^ Program in Public Health, University of California, Irvine, CA, USA

***** denotes shared senior authorship

**Table of Contents**

Supplemental Table S1

Supplemental Table S2

Supplemental Table S3

Supplemental Table S4

Supplemental Table S5

Supplemental Table S6

Supplemental Figure S1

| **Supplemental Table S1: Distributions of each HAPs (µg/m^3^) among participants of the Nurses’ Health Study II.** | | | | | | | |
| --- | --- | --- | --- | --- | --- | --- | --- |
| **Mammary carcinogens** | **Mean** | **SD** | **Min** | **25th %** | **Median** | **75th %** | **Max** |
| 1,2-Dibromo-3-Chloropropane | 6.12E-06 | 1.20E-05 | 1.30E-06 | 1.30E-06 | 2.23E-06 | 7.32E-06 | 1.44E-03 |
| 1,3-Butadiene | 8.44E-02 | 6.03E-02 | 4.68E-04 | 5.00E-02 | 7.00E-02 | 1.10E-01 | 5.09E+00 |
| 1,4-Dioxane | 2.46E-04 | 3.26E-03 | 0.00E+00 | 0.00E+00 | 0.00E+00 | 5.96E-06 | 9.30E-01 |
| 2,4-Dinitrotoluene | 1.36E-05 | 5.39E-05 | 0.00E+00 | 1.66E-06 | 5.51E-06 | 1.32E-05 | 8.20E-03 |
| 2,4-Toluene Diisocyanate | 1.38E-04 | 1.84E-03 | 3.10E-09 | 1.38E-05 | 3.11E-05 | 6.34E-05 | 3.20E-01 |
| 2-Chloroacetophenone | 6.73E-07 | 4.35E-05 | 0.00E+00 | 0.00E+00 | 7.27E-08 | 2.68E-07 | 6.57E-03 |
| Acrylonitrile | 7.07E-03 | 1.23E-02 | 5.66E-04 | 3.29E-03 | 6.32E-03 | 9.35E-03 | 1.35E+00 |
| Benzene (including Benzene from Gasoline) | 1.34E+00 | 7.44E-01 | 1.00E-01 | 9.00E-01 | 1.00E+00 | 2.00E+00 | 4.00E+01 |
| Benzidine | 1.73E-07 | 1.77E-06 | 9.90E-09 | 9.90E-09 | 1.09E-08 | 5.76E-08 | 1.80E-04 |
| Carbon Tetrachloride | 6.10E-01 | 1.83E-03 | 6.10E-01 | 6.10E-01 | 6.10E-01 | 6.10E-01 | 8.20E-01 |
| Chloroprene | 3.52E-04 | 6.56E-03 | 0.00E+00 | 1.00E-06 | 2.96E-06 | 7.49E-06 | 6.50E-01 |
| Diesel Engine Emissions | 1.03E+00 | 8.21E-01 | 7.00E-03 | 6.20E-01 | 9.10E-01 | 1.23E+00 | 3.85E+01 |
| Ethylene Dibromide (Dibromoethane) | 6.20E-04 | 3.99E-04 | 1.10E-04 | 2.94E-04 | 4.77E-04 | 9.38E-04 | 5.55E-03 |
| Ethylene Dichloride (1,2-Dichloroethane) | 3.93E-03 | 4.20E-03 | 1.80E-03 | 2.70E-03 | 3.14E-03 | 4.09E-03 | 3.40E-01 |
| Ethylene Oxide | 6.21E-03 | 8.22E-03 | 4.60E-04 | 2.90E-03 | 5.25E-03 | 8.21E-03 | 7.40E-01 |
| Ethylidene Dichloride (1,1-Dichloroethane) | 1.28E-03 | 7.89E-03 | 0.00E+00 | 2.45E-05 | 1.56E-04 | 9.09E-04 | 9.30E-01 |
| Hydrazine | 2.20E-05 | 1.40E-03 | 1.30E-07 | 1.30E-07 | 5.82E-07 | 7.96E-06 | 3.30E-01 |
| Methylene Chloride (Dichloromethane) | 3.73E-01 | 3.87E-01 | 1.00E-01 | 2.00E-01 | 3.00E-01 | 4.00E-01 | 3.00E+01 |
| Nitrobenzene | 1.41E-05 | 2.16E-04 | 0.00E+00 | 2.89E-07 | 9.02E-07 | 2.30E-06 | 2.00E-02 |
| O-Toluidine | 2.87E-06 | 3.11E-05 | 0.00E+00 | 1.30E-08 | 7.03E-08 | 3.47E-07 | 2.11E-03 |
| Propylene Dichloride (1,2-Dichloropropane) | 1.99E-03 | 2.28E-03 | 4.61E-04 | 6.98E-04 | 1.06E-03 | 2.06E-03 | 1.20E-01 |
| Propylene Oxide | 4.54E-04 | 2.45E-03 | 0.00E+00 | 4.90E-05 | 1.38E-04 | 3.51E-04 | 2.50E-01 |
| Styrene | 5.25E-02 | 2.50E-01 | 9.71E-06 | 1.00E-02 | 3.00E-02 | 5.00E-02 | 5.00E+01 |
| Vinyl Chloride | 4.27E-03 | 1.95E-02 | 0.00E+00 | 1.35E-04 | 1.02E-03 | 4.07E-03 | 1.84E+00 |
| Vinylidene Chloride (1,1-Dichloroethylene) | 2.02E-04 | 7.69E-04 | 0.00E+00 | 4.22E-05 | 9.05E-05 | 1.89E-04 | 7.00E-02 |
| **Estrogen disruptors** |  |  |  |  |  |  |  |
| 4-Nitrophenol | 7.17E-05 | 1.18E-04 | 0.00E+00 | 1.80E-05 | 4.16E-05 | 8.34E-05 | 5.53E-03 |
| Arsenic Compounds (Inorganic) | 6.06E-04 | 4.75E-04 | 1.30E-04 | 3.73E-04 | 5.02E-04 | 7.87E-04 | 2.00E-02 |
| Biphenyl | 2.88E-04 | 4.25E-03 | 5.03E-09 | 2.79E-05 | 6.32E-05 | 1.71E-04 | 7.10E-01 |
| Bis(2-Ethylhexyl)Phthalate (DEHP) | 6.22E-03 | 9.39E-03 | 5.20E-03 | 5.49E-03 | 5.67E-03 | 5.95E-03 | 1.82E+00 |
| Dibutulphthalate | 5.41E-03 | 2.31E-02 | 1.21E-08 | 3.20E-05 | 8.62E-05 | 3.70E-04 | 2.95E+00 |
| Diesel Engine Emissions | 1.03E+00 | 8.21E-01 | 7.00E-03 | 6.20E-01 | 9.10E-01 | 1.23E+00 | 3.85E+01 |
| Dimethyl Formamide | 2.07E-03 | 4.53E-03 | 0.00E+00 | 1.91E-04 | 1.14E-03 | 2.62E-03 | 7.80E-01 |
| Selenium Compounds | 1.85E-04 | 3.97E-04 | 9.65E-10 | 2.78E-05 | 8.83E-05 | 2.37E-04 | 3.00E-02 |
| Styrene | 5.25E-02 | 2.50E-01 | 9.71E-06 | 1.00E-02 | 3.00E-02 | 5.00E-02 | 5.00E+01 |

| **Supplemental Table S2: Associations of increasing quartiles of each HAPs exposure on risk of incident invasive breast cancer 1989-2011 among premenopausal members of the Nurses’ Health Study II cohort. The top row of each pair presents the hazard ratios (HR) and 95 % confidence intervals (95%CI) from basic models and the bottom row presents those from multivariable models*.** | | | | | | | | | |
| --- | --- | --- | --- | --- | --- | --- | --- | --- | --- |
|  | **Quartile 1** | | **Quartile 2** | | **Quartile 3** | | **Quartile 4** | |  |
| **Hazardous Air Pollutant** | Cases | HR  (95% CI) | Cases | HR  (95% CI) | Cases | HR  (95% CI) | Cases | HR  (95% CI) | P for Trend |
| **Mammary Carcinogens** |  |  |  |  |  |  |  |  |  |
| 1,2-Dibromo-3-Chloropropane | 562 | Ref | 433 | 1.01 (0.89, 1.14) | 473 | 0.99  (0.88, 1.12) | 591 | 1.18  (1.05, 1.33)* | 0.002* |
|  |  | Ref |  | 1.00 (0.85, 1.17) |  | 1.00  (0.84, 1.20) |  | 1.16  (0.97, 1.39) | 0.019* |
| 1,3-Butadiene | 422 | Ref | 587 | 0.98 (0.87, 1.11) | 472 | 1.07  (0.93, 1.22) | 578 | 1.11  (0.98, 1.26) | 0.037* |
|  |  | Ref |  | 0.92 (0.81, 1.05) |  | 0.97  (0.84, 1.12) |  | 1.02  (0.88, 1.17) | 0.376 |
| 1,4-Dioxane | 1114 | Ref | 435 | 0.96 (0.86, 1.07) | 510 | 0.97  (0.88, 1.08) |  |  | 0.777 |
|  |  | Ref |  | 0.93 (0.83, 1.05) |  | 0.96  (0.85, 1.09) |  |  | 0.818 |
| 2,4-Dinitrotoluene | 485 | Ref | 511 | 1.08 (0.95, 1.22) | 537 | 1.12  (0.99, 1.27) | 526 | 1.11  (0.98, 1.25) | 0.181 |
|  |  | Ref |  | 1.10 (0.93, 1.30) |  | 1.10  (0.93, 1.31) |  | 1.07  (0.90, 1.27) | 0.958 |
| 2,4-Toluene Diisocyanate | 475 | Ref | 493 | 1.00 (0.88, 1.13) | 578 | 1.17  (1.04, 1.33)* | 513 | 1.08  (0.96, 1.23) | 0.238 |
|  |  | Ref |  | 0.95 (0.83, 1.08) |  | 1.09  (0.96, 1.24) |  | 1.04  (0.91, 1.19) | 0.396 |
| 2-Chloroacetophenone | 608 | Ref | 447 | 1.03 (0.91, 1.17) | 519 | 1.00  (0.89, 1.13) | 485 | 0.95  (0.84, 1.07) | 0.255 |
|  |  | Ref |  | 0.97 (0.84, 1.13) |  | 0.93  (0.81, 1.07) |  | 0.90  (0.78, 1.04) | 0.133 |
| Acrylonitrile | 513 | Ref | 519 | 0.97 (0.86, 1.10) | 524 | 0.99  (0.87, 1.11) | 503 | 0.95  (0.84, 1.08) | 0.545 |
|  |  | Ref |  | 0.95 (0.84, 1.08) |  | 0.99  (0.87, 1.13) |  | 0.96  (0.83, 1.11) | 0.756 |
| Benzene (Including Benzene From Gasoline) | 466 | Ref | 522 | 1.09 (0.96, 1.23) | 516 | 1.05  (0.93, 1.19) | 555 | 1.16  (1.02, 1.31)* | 0.033* |
|  |  | Ref |  | 1.03 (0.90, 1.17) |  | 0.97  (0.85, 1.10) |  | 1.07  (0.93, 1.22) | 0.418 |
| Benzidine | 909 | Ref | 111 | 0.98 (0.80, 1.19) | 552 | 1.04  (0.93, 1.15) | 487 | 0.97  (0.87, 1.08) | 0.457 |
|  |  | Ref |  | 0.94 (0.77, 1.15) |  | 0.96  (0.84, 1.10) |  | 0.92  (0.80, 1.06) | 0.317 |
| Carbon Tetrachloride | 2042 | Ref | 17 | 1.59 (0.98, 2.56) |  |  |  |  |  |
|  |  | Ref |  | 1.56 (0.96, 2.53) |  |  |  |  |  |
| Chloroprene | 494 | Ref | 520 | 1.06 (0.94, 1.20) | 557 | 1.13  (1.00, 1.28)* | 488 | 1.01  (0.89, 1.15) | 0.714 |
|  |  | Ref |  | 1.04 (0.90, 1.19) |  | 1.07  (0.93, 1.23) |  | 0.95  (0.82, 1.10) | 0.196 |
| Diesel Engine Emissions^a^ | 470 | Ref | 503 | 1.05 (0.93, 1.19) | 524 | 1.08  (0.96, 1.23) | 562 | 1.17  (1.03, 1.32)* | 0.011* |
|  |  | Ref |  | 1.03 (0.90, 1.17) |  | 1.03  (0.90, 1.17) |  | 1.13  (0.98, 1.29) | 0.080 |
| Ethylene Dibromide (Dibromoethane) | 567 | Ref | 507 | 0.90 (0.80, 1.02) | 474 | 0.89  (0.79, 1.00) | 511 | 0.93  (0.82, 1.05) | 0.496 |
|  |  | Ref |  | 0.90 (0.79, 1.02) |  | 0.90  (0.78, 1.05) |  | 0.93  (0.80, 1.08) | 0.870 |
| Ethylene Dichloride (1,2-Dichloroethane) | 508 | Ref | 509 | 0.99 (0.87, 1.12) | 507 | 0.99  (0.88, 1.12) | 535 | 1.07  (0.94, 1.21) | 0.197 |
|  |  | Ref |  | 0.96 (0.84, 1.08) |  | 0.95  (0.84, 1.08) |  | 1.03  (0.90, 1.17) | 0.450 |
| Ethylene Oxide | 505 | Ref | 482 | 0.98 (0.86, 1.11) | 540 | 1.07  (0.95, 1.21) | 532 | 1.07  (0.95, 1.21) | 0.130 |
|  |  | Ref |  | 0.99 (0.87, 1.12) |  | 1.05  (0.92, 1.19) |  | 1.05  (0.93, 1.19) | 0.301 |
| Ethylidene Dichloride (1,1-Dichloroethane) | 477 | Ref | 529 | 1.10 (0.97, 1.24) | 533 | 1.09  (0.96, 1.23) | 520 | 1.06  (0.93, 1.20) | 0.985 |
|  |  | Ref |  | 1.09 (0.97, 1.24) |  | 1.05  (0.93, 1.19) |  | 1.03  (0.89, 1.20) | 0.784 |
| Hydrazine | 600 | Ref | 387 | 1.03 (0.91, 1.17) | 602 | 1.21  (1.08, 1.35)* | 470 | 0.97  (0.86, 1.10) | 0.092 |
|  |  | Ref |  | 1.04 (0.91, 1.19) |  | 1.17  (1.04, 1.31)* |  | 0.95  (0.84, 1.07) | 0.047* |
| Methylene Chloride (Dichloromethane) | 445 | Ref | 541 | 1.10 (0.97, 1.24) | 524 | 1.09  (0.96, 1.24) | 549 | 1.14  (1.00, 1.29)* | 0.083 |
|  |  | Ref |  | 1.03 (0.91, 1.17) |  | 1.00  (0.88, 1.15) |  | 1.08  (0.93, 1.25) | 0.328 |
| Nitrobenzene | 492 | Ref | 487 | 1.00 (0.89, 1.14) | 530 | 1.08  (0.96, 1.22) | 550 | 1.13  (1.00, 1.28)* | 0.030* |
|  |  | Ref |  | 0.98 (0.85, 1.13) |  | 1.02  (0.89, 1.18) |  | 1.06  (0.92, 1.22) | 0.264 |
| O-Toluidine | 512 | Ref | 500 | 0.99 (0.87, 1.12) | 552 | 1.08  (0.96, 1.22) | 495 | 1.00  (0.88, 1.13) | 0.759 |
|  |  | Ref |  | 0.94 (0.82, 1.08) |  | 1.01  (0.88, 1.15) |  | 0.93  (0.81, 1.07) | 0.383 |
| Propylene Dichloride (1,2-Dichloropropane) | 482 | Ref | 505 | 1.00 (0.88, 1.13) | 498 | 0.99  (0.87, 1.12) | 574 | 1.11  (0.98, 1.25) | 0.031* |
|  |  | Ref |  | 0.97 (0.85, 1.10) |  | 0.96  (0.83, 1.10) |  | 1.02  (0.85, 1.22) | 0.474 |
| Propylene Oxide | 485 | Ref | 534 | 1.11 (0.98, 1.25) | 507 | 1.05  (0.93, 1.19) | 533 | 1.11  (0.98, 1.26) | 0.235 |
|  |  | Ref |  | 1.08 (0.95, 1.23) |  | 1.01  (0.88, 1.16) |  | 1.07  (0.93, 1.22) | 0.602 |
| Styrene^a^ | 632 | Ref | 385 | 0.99 (0.87, 1.12) | 475 | 0.98  (0.87, 1.10) | 567 | 1.01  (0.90, 1.13) | 0.808 |
|  |  | Ref |  | 0.94 (0.83, 1.07) |  | 0.94  (0.83, 1.06) |  | 0.99  (0.88, 1.11) | 0.852 |
| Vinyl Chloride | 470 | Ref | 509 | 1.04 (0.92, 1.18) | 547 | 1.11  (0.98, 1.26) | 533 | 1.07  (0.95, 1.21) | 0.466 |
|  |  | Ref |  | 1.03 (0.91, 1.17) |  | 1.06  (0.93, 1.21) |  | 1.00  (0.86, 1.16) | 0.702 |
| Vinylidene Chloride (1,1-Dichloroethylene) | 482 | Ref | 532 | 1.09 (0.96, 1.23) | 521 | 1.06  (0.94, 1.20) | 524 | 1.09  (0.96, 1.23) | 0.390 |
|  |  | Ref |  | 1.04 (0.92, 1.18) |  | 1.00  (0.87, 1.13) |  | 1.01  (0.89, 1.16) | 0.967 |
| **Estrogen Disruptors** |  |  |  |  |  |  |  |  |  |
| Diesel Engine Emissions^a^ | 470 | Ref | 503 | 1.05 (0.93, 1.19) | 524 | 1.08  (0.96, 1.23) | 562 | 1.17  (1.03, 1.32)* | 0.011* |
|  |  | Ref |  | 1.03 (0.90, 1.17) |  | 1.03  (0.90, 1.17) |  | 1.13  (0.98, 1.29) | 0.080 |
| Arsenic Compounds (Inorganic) | 469 | Ref | 514 | 1.05 (0.93, 1.19) | 554 | 1.13  (1.00, 1.28)* | 522 | 1.07  (0.94, 1.21) | 0.246 |
|  |  | Ref |  | 0.97 (0.85, 1.11) |  | 1.03  (0.90, 1.17) |  | 0.99  (0.87, 1.13) | 0.976 |
| Biphenyl | 463 | Ref | 524 | 1.11 (0.98, 1.26) | 558 | 1.20  (1.06, 1.36)* | 514 | 1.10  (0.97, 1.24) | 0.600 |
|  |  | Ref |  | 1.07 (0.94, 1.22) |  | 1.12  (0.99, 1.28) |  | 1.01  (0.88, 1.15) | 0.492 |
| Bis(2-Ethylhexyl)Phthalate (Dehp) | 508 | Ref | 508 | 1.01 (0.89, 1.15) | 535 | 1.07  (0.94, 1.21) | 508 | 1.03  (0.91, 1.17) | 0.641 |
|  |  | Ref |  | 1.00 (0.88, 1.14) |  | 1.03  (0.91, 1.17) |  | 1.03  (0.90, 1.16) | 0.675 |
| Dibutulphthalate | 461 | Ref | 482 | 1.01 (0.89, 1.15) | 619 | 1.32  (1.17, 1.49)* | 497 | 1.08  (0.95, 1.23) | 0.695 |
|  |  | Ref |  | 0.97 (0.85, 1.10) |  | 1.26  (1.10, 1.43)* |  | 1.06  (0.93, 1.21) | 0.986 |
| Dimethyl Formamide | 482 | Ref | 481 | 1.00 (0.88, 1.13) | 533 | 1.09  (0.97, 1.24) | 563 | 1.18  (1.04, 1.33)* | 0.002* |
|  |  | Ref |  | 0.99 (0.87, 1.13) |  | 1.05  (0.92, 1.20) |  | 1.13  (0.99, 1.29) | 0.031* |
| 4-Nitrophenol | 465 | Ref | 504 | 1.05 (0.92, 1.19) | 527 | 1.09  (0.96, 1.24) | 563 | 1.17  (1.04, 1.33)* | 0.009* |
|  |  | Ref |  | 1.00 (0.88, 1.14) |  | 1.02  (0.89, 1.16) |  | 1.11  (0.97, 1.27) | 0.076 |
| Selenium Compounds | 479 | Ref | 515 | 1.09 (0.96, 1.23) | 550 | 1.13  (1.00, 1.28)* | 515 | 1.05  (0.93, 1.19) | 0.813 |
|  |  | Ref |  | 1.09 (0.96, 1.23) |  | 1.08  (0.95, 1.22) |  | 0.99  (0.86, 1.14) | 0.473 |
| Styrene^a^ | 632 | Ref | 385 | 0.99 (0.87, 1.12) | 475 | 0.98  (0.87, 1.10) | 567 | 1.01  (0.90, 1.13) | 0.808 |
|  |  | Ref |  | 1.08 (0.95, 1.23) |  | 1.01  (0.88, 1.16) |  | 1.07  (0.93, 1.22) | 0.602 |
| **Note:** All models adjusted for age, calendar period, race, family history of breast cancer, history of aspiration or biopsy confirmed benign breast disease, age at menarche, parity and age at first birth, oral contraception use, recent mammogram, height, BMI at age 18, difference between current BMI and BMI at age 18, smoking status, physical activity, overall diet quality (including alcohol consumption), alcohol consumption at age 15 and age 18, shift work, individual-level SES (marital status, living arrangements, household income), area-level SES (Census tract median home value and median income), and Census region of residence | | | | | | | | | |
| ^a^ Diesel exhaust and Styrene are both potential estrogen disruptors and mammary carcinogens  Grey boxes indicate HAPs without enough variability to create quartiles; ***** indicates p-values<0.05 | | | | | | | | | |

| **Supplemental Table S3: Associations of increasing quartiles of each HAPs exposure on risk of incident invasive breast cancer 1989-2011 among never smokers in the Nurses’ Health Study II cohort. The top row of each pair presents the hazard ratios (HR) and 95 % confidence intervals (95%CI) from basic^a^ models and the bottom row presents those from multivariable models^b^.** | | | | | | | | | | | | | | | | | | | | | | | | | | | | | | | | |  | | |  |  |
| --- | --- | --- | --- | --- | --- | --- | --- | --- | --- | --- | --- | --- | --- | --- | --- | --- | --- | --- | --- | --- | --- | --- | --- | --- | --- | --- | --- | --- | --- | --- | --- | --- | --- | --- | --- | --- | --- |
|  | **Quartile 1** | | | | | | **Quartile 2** | | | | | | | **Quartile 3** | | | | | | | **Quartile 4** | | | | | | |  | | | | |  | | |  |  |
| **Hazardous Air Pollutant** | **cases** | | **HR (95%CI)** | | | | **cases** | | | **HR**  **(95% CI)** | | | | **cases** | | | **Hazardous Air Pollutant** | | | | **cases** | | | **HR (95%CI)** | | | | **p for trend** | | | | |  | | |  |  |
| **Mammary Carcinogens** |  | |  | | | |  | | |  | | | |  | | |  | | | |  | | |  | | | |  | | | | |  | | |  |  |
| 1,2-Dibromo-3-Chloropropane | 636 | | Ref | | | | 439 | | | 0.97 (0.86, 1.10) | | | | 468 | | | 0.94 (0.83, 1.06) | | | | 522 | | | 1.01  (0.98, 1.23) | | | | 0.069 | | | | |  | | |  |  |
|  |  |  | Ref | | | |  |  |  | 0.95 (0.82, 1.10) | | | |  |  |  | 0.94 (0.79, 1.11) | | | |  |  |  | 1.08 (0.91,1.29) | | | | 0.057 | | | | |  | | |  |  |
| 1,3-Butadiene | 459 | | Ref | | | | 643 | | | 1.04 (0.93, 1.18) | | | | 461 | | | 1.07 (0.94, 1.22) | | | | 502 | | | 1.01  (0.89, 1.14) | | | | 1.00 | | | | |  | | |  |  |
|  |  |  | Ref | | | |  |  |  | 0.98 (0.87, 1.11) | | | |  |  |  | 0.99 (0.86, 1.14) | | | |  |  |  | 0.96  (0.83, 1.10) | | | | 0.546 | | | | |  | | |  |  |
| 1,4-Dioxane | 1111 | | Ref | | | | 431 | | | 0.99 (0.89, 1.11) | | | | 523 | | | 0.97 (0.87, 1.07) | | | |  | | |  | | | | 0.503 | | | | |  | | |  |  |
|  |  |  | Ref | | | |  |  |  | 0.98 (0.88, 1.10) | | | |  |  |  | 0.92 (0.81, 1.05) | | | |  | | |  | | | | 0.233 | | | | |  | | |  |  |
| 2,4-Dinitrotoluene | 533 | | Ref | | | | 534 | | | 1.10 (0.97, 1.24) | | | | 521 | | | 1.07 (0.95, 1.21) | | | | 477 | | | 1.06 (0.94,1.20) | | | | 0.598 | | | | |  | | |  |  |
|  |  |  | Ref | | | |  |  |  | 1.12 (0.95, 1.31) | | | |  |  |  | 1.06 (0.90, 1.25) | | | |  |  |  | 1.06  (0.90, 1.26) | | | | 0.947 | | | | |  | | |  |  |
| 2,4-Toluene Diisocyanate | 543 | | Ref | | | | 488 | | | 0.93 (0.82, 1.05) | | | | 530 | | | 1.07 (0.95, 1.21) | | | | 504 | | | 0.97  (0.86, 1.10) | | | | 0.849 | | | | |  | | |  |  |
|  |  |  | Ref | | | |  |  |  | 0.88 (0.77, 1.00) | | | |  |  |  | 1.00 (0.88, 1.13) | | | |  |  |  | 0.93  (0.81, 1.06) | | | | 0.605 | | | | |  | | |  |  |
| 2-Chloroacetophenone | 622 | | Ref | | | | 456 | | | 1.03 (0.91, 1.16) | | | | 488 | | | 0.95 (0.84, 1.07) | | | | 459 | | | 0.87  (0.77, 0.98)* | | | | 0.007* | | | | |  | | |  |  |
|  |  |  | Ref | | | |  |  |  | 0.96 (0.83, 1.11) | | | |  |  |  | 0.89 (0.77, 1.02) | | | |  |  |  | 0.81  (0.70, 0.93)* | | | | 0.001* | | | | |  | | |  |  |
| Acrylonitrile | 521 | | Ref | | | | 497 | | | 1.00 (0.88, 1.13) | | | | 509 | | | 0.99 (0.88, 1.12) | | | | 538 | | | 0.99  (0.88, 1.12) | | | | 0.870 | | | | |  | | |  |  |
|  |  |  | Ref | | | |  |  |  | 1.01 (0.89, 1.14) | | | |  |  |  | 0.99 (0.87, 1.14) | | | |  |  |  | 0.98  (0.85, 1.14) | | | | 0.812 | | | | |  | | |  |  |
| Benzene (Including Benzene From Gasoline) | 524 | | Ref | | | | 551 | | | 1.08 (0.96, 1.22) | | | | 497 | | | 1.02 (0.90, 1.15) | | | | 493 | | | 1.05  (0.93, 1.19) | | | | 0.613 | | | | |  | | |  |  |
|  |  |  | Ref | | | |  |  |  | 1.03 (0.91, 1.16) | | | |  |  |  | 0.96 (0.84, 1.09) | | | |  |  |  | 1.01  (0.88, 1.15) | | | | 0.934 | | | | |  | | |  |  |
| Benzidine | 956 | | Ref | | | | 105 | | | 0.92 (0.75, 1.12) | | | | 505 | | | 0.97 (0.87, 1.08) | | | | 499 | | | 0.96  (0.86, 1.07) | | | | 0.632 | | | | |  | | |  |  |
|  |  |  | Ref | | | |  |  |  | 0.91 (0.74, 1.12) | | | |  |  |  | 0.95 (0.83, 1.09) | | | |  |  |  | 0.91  (0.79, 1.04) | | | | 0.278 | | | | |  | | |  |  |
| Carbon Tetrachloride | 2054 | | Ref | | | | 11 | | | 1.10 (0.61, 2.01) | | | |  | | |  | | | |  | | |  | | | |  | | | | |  | | |  |  |
|  |  |  | Ref | | | |  |  |  | 1.09 (0.60, 1.99) | | | |  | | |  | | | |  | | |  | | | |  | | | | |  | | |  |  |
| Chloroprene | 556 | | Ref | | | | 517 | | | 1.00 (0.89, 1.13) | | | | 530 | | | 1.04 (0.93, 1.18) | | | | 462 | | | 0.95  (0.84, 1.07) | | | | 0.310 | | | | |  | | |  |  |
|  |  |  | Ref | | | |  |  |  | 0.97 (0.84, 1.11) | | | |  |  |  | 0.97 (0.85, 1.12) | | | |  |  |  | 0.91  (0.79, 1.05) | | | | 0.208 | | | | |  | | |  |  |
| Diesel Engine Emissions^a^ | 512 | | Ref | | | | 510 | | | 1.00 (0.88, 1.13) | | | | 514 | | | 1.05 (0.93, 1.19) | | | | 529 | | | 1.07  (0.95, 1.21) | | | | 0.181 | | | | |  | | |  |  |
|  |  | | Ref | | | |  | | | 0.96 (0.85, 1.10) | | | |  | | | 1.00 (0.87, 1.14) | | | |  | | | 1.05  (0.91, 1.20) | | | | 0.401 | | | | |  | | |  |  |
| Ethylene Dibromide (Dibromoethane) | 551 | | Ref | | | | 471 | | | 0.89 (0.78, 1.00) | | | | 528 | | | 0.93 (0.83, 1.05) | | | | 515 | | | 0.92  (0.81, 1.04) | | | | 0.476 | | | | |  | | |  |  |
|  |  |  | Ref | | | |  |  |  | 0.87 (0.77, 1.00) | | | |  |  |  | 0.88 (0.76, 1.02) | | | |  |  |  | 0.86  (0.74, 1.01) | | | | 0.280 | | | | |  | | |  |  |
| Ethylene Dichloride (1,2-Dichloroethane) | 548 | | Ref | | | | 503 | | | 0.94 (0.84, 1.06) | | | | 496 | | | 0.96 (0.85, 1.08) | | | | 518 | | | 0.99  (0.88, 1.12) | | | | 0.862 | | | | |  | | |  |  |
|  |  |  | Ref | | | |  |  |  | 0.93 (0.82, 1.05) | | | |  |  |  | 0.94 (0.83, 1.06) | | | |  |  |  | 0.97  (0.85, 1.11) | | | | 0.975 | | | | |  | | |  |  |
| Ethylene Oxide | 521 | | Ref | | | | 517 | | | 1.01 (0.89, 1.14) | | | | 523 | | | 1.05 (0.93, 1.19) | | | | 504 | | | 1.04  (0.92, 1.18) | | | | 0.409 | | | | |  | | |  |  |
|  |  |  | Ref | | | |  |  |  | 1.02 (0.90, 1.15) | | | |  |  |  | 1.04 (0.92, 1.18) | | | |  |  |  | 1.05  (0.92, 1.19) | | | | 0.468 | | | | |  | | |  |  |
| Ethylidene Dichloride (1,1-Dichloroethane) | 521 | | Ref | | | | 530 | | | 1.05 (0.93, 1.19) | | | | 489 | | | 1.01 (0.89, 1.14) | | | | 534 | | | 1.01  (0.90, 1.15) | | | | 0.888 | | | | |  | | |  |  |
|  |  |  | Ref | | | |  |  |  | 1.03 (0.91, 1.17) | | | |  |  |  | 0.98 (0.86, 1.12) | | | |  |  |  | 0.97  (0.86, 1.12) | | | | 0.510 | | | | |  | | |  |  |
| Hydrazine | 652 | | Ref | | | | 399 | | | 1.00 (0.88 1.13) | | | | 549 | | | 1.11 (0.99, 1.24) | | | | 465 | | | 0.92  (0.82, 1.04) | | | | 0.051 | | | | |  | | |  |  |
|  |  |  | Ref | | | |  |  |  | 1.01 (0.89, 1.15) | | | |  |  |  | 1.10 (0.98, 1.24) | | | |  |  |  | 0.93  (0.82, 1.04) | | | | 0.051 | | | | |  | | |  |  |
| Methylene Chloride (Dichloromethane) | 509 | | Ref | | | | 549 | | | 1.02 (0.90 1.15) | | | | 501 | | | 1.02 (0.90, 1.15) | | | | 506 | | | 1.00  (0.89, 1.14) | | | | 0.969 | | | | |  | | |  |  |
|  |  |  | Ref | | | |  |  |  | 0.96  (0.85 ,1.09) | | | |  |  |  | 0.95  (0.83, 1.08) | | | |  |  |  | 0.96  (0.83, 1.11) | | | | 0.630 | | | | |  | | |  |  |
| Nitrobenzene | 542 | | Ref | | | | 514 | | | 1.00 (0.89, 1.13) | | | | 536 | | | 1.07 (0.95, 1.20) | | | | 473 | | | 1.02  (0.90, 1.15) | | | | 0.893 | | | | |  | | |  |  |
|  |  |  | Ref | | | |  |  |  | 0.98 (0.85, 1.12) | | | |  |  |  | 1.01  (0.88, 1.16) | | | |  |  |  | 0.98  (0.85, 1.13) | | | | 0.821 | | | | |  | | |  |  |
| O-Toluidine | 574 | | Ref | | | | 483 | | | 0.89 (0.79, 1.01) | | | | 544 | | | 1.03 (0.91, 1.16) | | | | 464 | | | 0.92  (0.82, 1.04) | | | | 0.405 | | | | |  | | |  |  |
|  |  |  | Ref | | | |  |  |  | 0.84 (0.73, 0.96) | | | |  |  |  | 0.95 (0.83, 1.09) | | | |  |  |  | 0.87  (0.75, 1.00) | | | | 0.285 | | | | |  | | |  |  |
| Propylene Dichloride (1,2-Dichloropropane) | 551 | | Ref | | | | 519 | | | 0.92 (0.81, 1.04) | | | | 519 | | | 0.96 (0.85, 1.08) | | | | 476 | | | 0.95  (0.84, 0.79)* | | | | 0.884 | | | | |  | | |  |  |
|  |  |  | Ref | | | |  |  |  | 0.89 (0.78, 1.01) | | | |  |  |  | 0.95 (0.83, 1.09) | | | |  |  |  | 0.94  (0.79, 1.11) | | | | 0.797 | | | | |  | | |  |  |
| Propylene Oxide | 521 | | Ref | | | | 526 | | | 1.07 (0.95, 1.21) | | | | 519 | | | 1.07 (0.95, 1.21) | | | | 499 | | | 1.07  (0.95, 1.21) | | | | 0.431 | | | | |  | | |  |  |
|  |  |  | Ref | | | |  |  |  | 1.05 (0.92, 1.20) | | | |  |  |  | 1.05 (0.92, 1.21) | | | |  |  |  | 1.05  (0.92, 1.21) | | | | 0.662 | | | | |  | | |  |  |
| Styrene^a^ | 663 | | Ref | | | | 374 | | | 0.98 (0.86, 1.11) | | | | 458 | | | 0.96 (0.85, 1.09) | | | | 570 | | | 1.01  (0.90, 1.13) | | | | 0.812 | | | | |  | | |  |  |
|  |  |  | Ref | | | |  |  |  | 0.94 (0.83, 1.07) | | | |  |  |  | 0.93 (0.82, 1.05) | | | |  |  |  | 1.00  (0.89, 1.12) | | | | 0.775 | | | | |  | | |  |  |
| Vinyl Chloride | 529 | | Ref | | | | 515 | | | 0.99 (0.87, 1.12) | | | | 540 | | | 1.06 (0.94, 1.20) | | | | 481 | | | 0.98  (0.86, 1.11) | | | | 0.667 | | | | |  | | |  |  |
|  |  |  | Ref | | | |  |  |  | 0.97  (0.85, 1.10) | | | |  |  |  | 1.02 (0.90, 1.16) | | | |  |  |  | 0.95  (0.82, 1.10) | | | | 0.531 | | | | |  | | |  |  |
| Vinylidene Chloride (1,1-Dichloroethylene) | 532 | | Ref | | | | 546 | | | 1.06 (0.94, 1.19) | | | | 504 | | | 1.01 (0.90, 1.14) | | | | 483 | | | 1.01  (0.89, 1.14) | | | | 0.847 | | | | |  | | |  |  |
|  |  |  | Ref | | | |  |  |  | 1.01 (0.89, 1.14) | | | |  |  |  | 0.96  (0.85, 1.09) | | | |  |  |  | 0.97  (0.85, 1.11) | | | | 0.625 | | | | |  | | |  |  |
| **Estrogen Disruptors** |  | | |  | | |  | | | | |  | |  | | | | |  | |  | | | | | |  | |  | | |  |  |  |  |  |  |
| Diesel Engine Emissions^a^ | 512 | | | | Ref | | | 510 | | | 1.00 (0.88, 1.13) | | | | 514 | | | 1.05 (0.93, 1.19) | | | | 529 | | | 1.07  (0.95, 1.21) | | | | | 0.181 | | | |  | | |  |
|  |  |  |  |  | Ref | | |  |  |  | 0.96 (0.85, 1.10) | | | |  |  |  | 1.00 (0.87, 1.14) | | | |  |  |  | 1.05  (0.91, 1.20) | | | | | 0.401 | | | |  | | |  |
| Arsenic Compounds (Inorganic) | 532 | | | | Ref | | | 502 | | | 0.99 (0.88, 1.12) | | | | 546 | | | 1.08 (0.96, 1.22) | | | | 485 | | | 0.98  (0.86, 1.11) | | | | | 0.962 | | | |  | | |  |
|  |  |  |  |  | Ref | | |  |  |  | 0.94 (0.83, 1.07) | | | |  |  |  | 1.01 (0.89, 1.14) | | | |  |  |  | 0.93  (0.82, 1.06) | | | | | 0.424 | | | |  | | |  |
| Biphenyl | 518 | | | | Ref | | | 550 | | | 1.10 (0.97, 1.24) | | | | 522 | | | 1.12 (0.99, 1.26) | | | | 475 | | | 1.02  (0.90, 1.15) | | | | | 0.600 | | | |  | | |  |
|  |  |  |  |  | Ref | | |  |  |  | 1.06 (0.93, 1.19) | | | |  |  |  | 1.07 (0.94, 1.21) | | | |  |  |  | 0.98  (0.86, 1.12) | | | | | 0.407 | | | |  | | |  |
| Bis(2-Ethylhexyl)Phthalate (Dehp) | 526 | | | | Ref | | | 489 | | | 0.94 (0.83, 1.07) | | | | 546 | | | 1.10 (0.97, 1.24) | | | | 504 | | | 0.99  (0.88, 1.12) | | | | | 0.933 | | | |  | | |  |
|  |  |  |  |  | Ref | | |  |  |  | 0.94 (0.82, 1.07) | | | |  |  |  | 1.09 (0.96, 1.24) | | | |  |  |  | 0.99  (0.87, 1.12) | | | | | 0.914 | | | |  | | |  |
| Dibutulphthalate | 527 | | | | Ref | | | 483 | | | 0.94 (0.83, 1.07) | | | | 498 | | | 1.05 (0.93, 1.18) | | | | 557 | | | 1.07  (0.95, 1.21) | | | | | 0.119 | | | |  | | |  |
|  |  | | | | Ref | | |  |  |  | 0.90 (0.79, 1.02) | | | |  |  |  | 1.00 (0.88, 1.14) | | | |  |  |  | 1.05  (0.93, 1.19) | | | | | 0.103 | | | |  | | |  |
| Dimethyl Formamide | 534 | | | | Ref | | | 525 | | | 0.99 (0.88, 1.12) | | | | 517 | | | 1.04 (0.92, 1.17) | | | | 489 | | | 1.07  (0.95, 1.21) | | | | | 0.178 | | | |  | | |  |
|  |  |  |  |  | Ref | | |  |  |  | 1.00 (0.88, 1.13) | | | |  |  |  | 1.02 (0.90, 1.16) | | | |  |  |  | 1.08  (0.94, 1.23) | | | | | 0.190 | | | |  | | |  |
| 4-Nitrophenol | 535 | | | | Ref | | | 518 | | | 1.00 (0.88, 1.13) | | | | 506 | | | 1.02 (0.91, 1.16) | | | | 506 | | | 1.04  (0.92, 1.18) | | | | | 0.458 | | | |  | | |  |
|  |  |  |  |  | Ref | | |  |  |  | 0.95 (0.83, 1.07) | | | |  |  |  | 0.96 (0.85, 1.10) | | | |  |  |  | 1.00  (0.88, 1.15) | | | | | 0.687 | | | |  | | |  |
| Selenium Compounds | 511 | | | | Ref | | | 534 | | | 1.05 (0.93, 1.19) | | | | 526 | | | 1.06 (0.93, 1.19) | | | | 494 | | | 0.99  (0.87, 1.12) | | | | | 0.597 | | | |  | | |  |
|  |  |  |  |  | Ref | | |  |  |  | 1.04 (0.92, 1.18) | | | |  |  |  | 1.03 (0.90, 1.17) | | | |  |  |  | 0.98  (0.85, 1.14) | | | | | 0.641 | | | |  | | |  |
| Styrene^a^ | | 663 | | | | Ref | | | 374 | | | | 0.98 (0.86, 1.11) | | | 458 | | | | 0.96 (0.85, 1.09) | | | 570 | | | 1.01  (0.90, 1.13) | | | | | 0.812 | | | |  | | |
|  |  | | | | Ref | | |  | | | 0.94 (0.83, 1.07) | | | |  | | | 0.93 (0.82, 1.05) | | | |  | | | 1.00  (0.89, 1.12) | | | | | 0.775 | | | |  | | |  |
| **^a^** Basic models are adjusted for time-varying current age and calendar period by stratification.  **^b^** All fully adjusted models are adjusted for time-varying current age and calendar period by stratification and include terms for race, family history of breast cancer, history of aspiration or biopsy confirmed benign breast disease, age at menarche, parity and age at first birth, menopausal status and postmenopausal hormone use, oral contraception use, recent mammogram, height, BMI at age 18, difference between current BMI and BMI at age 18, physical activity, overall diet quality (including alcohol consumption), alcohol consumption at age 15 and age 18, shift work, individual-level SES (marital status, living arrangements, household income), area-level SES (Census tract median home value and median income), and Census region of residence  ^a^ Diesel exhaust and Styrene are both potential estrogen disruptors and mammary carcinogens  Grey boxes indicate HAPs without enough variability to create quartiles; ***** indicates p-values<0.05 | | | | | | | | | | | | | | | | | | | | | | | | | | | | | | | | | | | | |  |

| **Supplemental Table S4: Associations of increasing quartiles of each HAPs exposure on risk of incident invasive breast cancer 1989-2011 among ever smokers in the Nurses’ Health Study II cohort. The top row of each pair presents the hazard ratios (HR) and 95 % confidence intervals (95%CI) from basic^a^ models and the bottom row presents those from multivariable models^b^.** | | | | | | | | | | | |  | | |  |  |
| --- | --- | --- | --- | --- | --- | --- | --- | --- | --- | --- | --- | --- | --- | --- | --- | --- |
|  | | **Quartile 1** | | **Quartile 2** | | **Quartile 3** | | **Quartile 4** | |  | | |  | | |  |
| **Hazardous Air Pollutant** | | **cases** | **HR (95%CI)** | **cases** | **HR**  **(95% CI)** | **cases** | **HR (95%CI)** | **cases** | **HR**  **(95%CI)** | | **p for trend** | | |  | | |
| **Mammary Carcinogens** | |  |  |  |  |  |  |  |  | |  | | |  | | |
| 1,2-Dibromo-3-Chloropropane | | 321 | Ref | 253 | 0.98  (0.83, 1.15) | 289 | 0.96  (0.82, 1.13) | 393 | 1.19  (1.02, 1.38)* | | 0.006* | | |  | | |
|  |  |  | Ref |  | 1.03  (0.83. 1.26) |  | 1.06  (0.83, 1.34) |  | 1.25  (0.99, 1.59) | | 0.014* | | |  | | |
| 1,3-Butadiene | | 247 | Ref | 350 | 1.00  (0.85, 1.18) | 315 | 1.23  (1.04, 1.45)* | 344 | 1.10  (0.93, 1.30) | | 0.121 | | |  | | |
|  |  |  | Ref |  | 0.93  (0.79, 1.11) |  | 1.12  (0.94, 1.34) |  | 1.01  (0.84, 1.21) | | 0.552 | | |  | | |
| 1,4-Dioxane | | 696 | Ref | 257 | 0.88  (0.76, 1.01) | 303 | 0.96  (0.83, 1.10) |  |  | | 0.899 | | |  | | |
|  |  |  | Ref |  | 0.86  (0.74, 1.00) |  | 0.94  (0.80, 1.11) |  |  | | 0.910 | | |  | | |
| 2,4-Dinitrotoluene | | 294 | Ref | 296 | 0.97  (0.83, 1.15) | 354 | 1.18  (1.01, 1.38) | 312 | 0.96  (0.82, 1.13) | | 0.614 | | |  | | |
|  |  |  | Ref |  | 0.98  (0.79, 1.21) |  | 1.15  (0.93, 1.42) |  | 0.91  (0.73, 1.13) | | 0.149 | | |  | | |
| 2,4-Toluene Diisocyanate | | 269 | Ref | 309 | 1.12  (0.95, 1.32) | 360 | 1.30  (1.11, 1.52) | 318 | 1.21  (1.03, 1.43) | | 0.075 | | |  | | |
|  |  |  | Ref |  | 1.08  (0.91, 1.28) |  | 1.21  (1.03, 1.43)* |  | 1.21  (1.01, 1.44)* | | 0.068 | | |  | | |
| 2-Chloroacetophenone | | 370 | Ref | 263 | 1.02  (0.87, 1.19) | 337 | 1.03  (0.88, 1.19) | 286 | 0.92  (0.79, 1.08) | | 0.222 | | |  | | |
|  |  |  | Ref |  | 0.99  (0.82, 1.20) |  | 0.98  (0.81, 1.17) |  | 0.90  (0.75, 1.08) | | 0.172 | | |  | | |
| Acrylonitrile | | 329 | Ref | 328 | 0.96  (0.83, 1.12) | 296 | 0.95  (0.81, 1.11) | 303 | 0.99  (0.85, 1.16) | | 0.851 | | |  | | |
|  |  |  | Ref |  | 0.92  (0.78, 1.07) |  | 0.94  (0.79, 1.11) |  | 0.99  (0.82, 1.19) | | 0.879 | | |  | | |
| Benzene (Including Benzene From Gasoline) | | 275 | Ref | 310 | 1.10  (0.94, 1.30) | 338 | 1.13  (0.96, 1.32) | 333 | 1.16  (0.98, 1.36) | | 0.089 | | |  | | |
|  |  |  | Ref |  | 1.04  (0.88, 1.23) |  | 1.03  (0.87, 1.22) |  | 1.08  (0.91, 1.28) | | 0.433 | | |  | | |
| Benzidine | | 508 | Ref | 70 | 1.05  (0.82, 1.35) | 361 | 1.12  (0.98, 1.28) | 317 | 1.06  (0.92, 1.22) | | 0.834 | | |  | | |
|  |  |  | Ref |  | 1.01  (0.78, 1.30) |  | 1.08  (0.91, 1.28) |  | 1.11  (0.93, 1.32) | | 0.404 | | |  | | |
| Carbon Tetrachloride | | 1249 | Ref | 7 | 0.87  (0.41, 1.84) |  |  |  |  | |  | | |  | | |
|  |  |  | Ref |  | 0.91  (0.43, 1.93) |  |  |  |  | |  | | |  | | |
| Chloroprene | | 301 | Ref | 303 | 1.00  (0.85, 1.18) | 368 | 1.20  (1.03, 1.40)* | 284 | 0.90  (0.77, 1.06) | | 0.087 | | |  | | |
|  |  |  | Ref |  | 1.01  (0.84, 1.21) |  | 1.17  (0.97, 1.39) |  | 0.86  (0.71, 1.04) | | 0.018* | | |  | | |
| Diesel Engine Emissions^a^ | | 283 | Ref | 318 | 1.18  (1.00, 1.38)* | 314 | 1.12  (0.95, 1.31) | 341 | 1.22  (1.04, 1.43)* | | 0.036* | | |  | | |
|  |  |  | Ref |  | 1.15  (0.98, 1.36) |  | 1.05  (0.88, 1.25) |  | 1.17  (0.98, 1.39) | | 0.168 | | |  | | |
| Ethylene Dibromide (Dibromoethane) | | 369 | Ref | 314 | 0.90  (0.77, 1.04) | 284 | 0.93  (0.79, 1.09) | 289 | 0.91  (0.78, 1.06) | | 0.449 | | |  | | |
|  |  |  | Ref |  | 0.90  (0.77, 1.06) |  | 1.00  ().83, 1.21) |  | 0.95  (0.78, 1.16) | | 0.876 | | |  | | |
| Ethylene Dichloride (1,2-Dichloroethane) | | 294 | Ref | 301 | 1.06  (0.90, 1.24) | 337 | 1.14  (0.98, 1.34) | 324 | 1.11  (0.95, 1.30) | | 0.228 | | |  | | |
|  |  |  | Ref |  | 1.03  (0.87, 1.21) |  | 1.11  (0.94, 1.30) |  | 1.07  (0.90, 1.26) | | 0.482 | | |  | | |
| Ethylene Oxide | | 312 | Ref | 274 | 0.93  (0.79, 1.09) | 346 | 1.14  (0.98, 1.33) | 324 | 1.06  (0.90, 1.23) | | 0.156 | | |  | | |
|  |  |  | Ref |  | 0.93  (0.79, 1.10) |  | 1.11  *0.95, 1.31) |  | 1.03  (0.88, 1.21) | | 0.334 | | |  | | |
| Ethylidene Dichloride (1,1-Dichloroethane) | | 296 | Ref | 308 | 1.01  (0.86, 1.18) | 322 | 1.04  (0.89, 1.22) | 330 | 1.13  (0.97, 1.33) | | 0.087 | | |  | | |
|  |  |  | Ref |  | 1.02  (0.87, 1.20) |  | 1.03  (0.87, 1.21) |  | 1.17  (0.97, 1.41) | | 0.080 | | |  | | |
| Hydrazine | | 369 | Ref | 229 | 1.02  (0.87, 1.21) | 381 | 1.18  (1.02, 1.37)* | 277 | 0.92  (0.78, 1.07) | | 0.040* | | |  | | |
|  |  |  | Ref |  | 1.03  (0.87, 1.23) |  | 1.13  (0.97, 1.32) |  | 0.90  (0.77, 1.06) | | 0.034* | | |  | | |
| Methylene Chloride (Dichloromethane) | | 270 | Ref | 300 | 1.00  (0.85, 1.18) | 348 | 1.20  (1.02, 1.40) | 338 | 1.17  (1.00, 1.38)* | | 0.023* | | |  | | |
|  |  |  | Ref |  | 0.93  (0.78, 1.10) |  | 1.10  (0.93, 1.30) |  | 1.13  (0.93, 1.36) | | 0.086 | | |  | | |
| Nitrobenzene | | 308 | Ref | 278 | 0.92  (0.78, 1.08) | 339 | 0.92  (0.94, 1.28) | 331 | 1.00  (0.86, 1.17) | | 0.777 | | |  | | |
|  |  |  | Ref |  | 0.90  (0.75, 1.08) |  | 1.05  (0.88, 1.26) |  | 0.94  (0.79, 1.13) | | 0.675 | | |  | | |
| O-Toluidine | | 310 | Ref | 314 | 1.02  (0.87, 1.20) | 322 | 1.02  (0.87, 1.19) | 310 | 0.96  (0.82, 1.13) | | 0.453 | | |  | | |
|  |  |  | Ref |  | 0.98  (0.82, 1.17) |  | 0.96  (0.80, 1.15) |  | 0.92  (0.77, 1.11) | | 0.389 | | |  | | |
| Propylene Dichloride (1,2-Dichloropropane) | | 279 | Ref | 299 | 1.07  (0.91, 1.27) | 288 | 0.99  (0.84, 1.17) | 390 | 1.18  (1.01, 1.38)* | | 0.019* | | |  | | |
|  |  |  | Ref |  | 1.05  (0.89, 1.25) |  | 0.96  (0.80, 1.16) |  | 1.13  (0.90, 1.42) | | 0.133 | | |  | | |
| Propylene Oxide | | 312 | Ref | 304 | 0.98  (0.83, 1.15) | 308 | 0.98  (0.83, 1.15) | 332 | 1.03  (0.88, 1.20) | | 0.431 | | |  | | |
|  |  |  | Ref |  | 0.94  (0.79, 1.12) |  | 0.92  (0.77, 1.09) |  | 0.97  (0.81, 1.15) | | 0.941 | | |  | | |
| Styrene^a^ | | 387 | Ref | 258 | 1.04  (0.88, 1.21) | 295 | 0.99  (0.85, 1.15) | 316 | 0.92  (0.80, 1.07) | | 0.812 | | |  | | |
|  |  |  | Ref |  | 1.00  (0.85, 1.18) |  | 0.96  (0.82, 1.12) |  | 0.93  (0.80, 1.08) | | 0.301 | | |  | | |
| Vinyl Chloride | | 266 | Ref | 294 | 1.05  (0.89, 1.24) | 359 | 1.26  (1.07, 1.48)* | 337 | 1.13  (0.96, 1.33) | | 0.667 | | |  | | |
|  |  |  | Ref |  | 1.02  (0.86, 1.21) |  | 1.19  (1.01, 1.41)* |  | 1.06  (0.88, 1.28) | | 0.947 | | |  | | |
| Vinylidene Chloride (1,1-Dichloroethylene) | | 287 | Ref | 314 | 1.10  (0.94, 1.29) | 351 | 1.20  (1.02, 1.40)* | 304 | 1.00  (0.85, 1.18) | | 0.847 | | |  | | |
|  |  |  | Ref |  | 1.06  (0.90, 1.25) |  | 1.13  (0.96, 1.33) |  | 0.94  (0.79, 1.11) | | 0.183 | | |  | | |
| **Estrogen Disruptors** | | |  |  |  |  |  |  |  | |  | | |  | | |
| Diesel Engine Emissions^a^ | 283 | | Ref | 318 | 1.18  (1.00, 1.38)* | 314 | 1.12  (0.95, 1.31) | 341 | 1.22  (1.04, 1.42)* | | 0.036* | | |  | | |
|  |  |  | Ref |  | 1.15  (0.98, 1.36) |  | 1.05  (0.88, 1.25) |  | 1.17  (0.98, 1.39) | | 0.168 | | |  | | |
| Arsenic Compounds (Inorganic) | 280 | | Ref | 353 | 1.17  (1.00, 1.37)* | 300 | 1.02  (0.86, 1.20) | 323 | 1.08  (0.92, 1.27) | | 0.710 | | |  | | |
|  |  |  | Ref |  | 1.08  (0.92, 1.28) |  | 0.92  (0.78, 1.10) |  | 0.99  (0.83, 1.17) | | 0.480 | | |  | | |
| Biphenyl | 283 | | Ref | 309 | 1.10  (0.93, 1.29) | 335 | 1.13  (0.96, 1.32) | 329 | 1.08  (0.92, 1.27) | | 0.705 | | |  | | |
|  |  |  | Ref |  | 1.07  (0.91, 1.26) |  | 1.07  (0.90, 1.26) |  | 0.99  (0.83, 1.18) | | 0.524 | | |  | | |
| Bis(2-Ethylhexyl)Phthalate (Dehp) | 307 | | Ref | 320 | 1.08  (0.92, 1.26) | 321 | 1.03  (0.88, 1.21) | 308 | 1.04  (0.88, 1.21) | | 0.856 | | |  | | |
|  |  |  | Ref |  | 1.08  (0.92, 1.28) |  | 0.99  (0.84, 1.17) |  | 1.05  (0.89, 1.23) | | 0.742 | | |  | | |
| Dibutulphthalate | 270 | | Ref | 307 | 1.13  (0.96, 1.33) | 404 | 1.41  (1.21, 1.65)* | 275 | 1.09  (0.92, 1.29) | | 0.258 | | |  | | |
|  |  |  | Ref |  | 1.08  (0.91, 1.28) |  | 1.36  (1.15, 1.61)* |  | 1.07  (0.90, 1.28) | | 0.425 | | |  | | |
| Dimethyl Formamide | 280 | | Ref | 288 | 1.04  (0.88, 1.23) | 350 | 1.22  (1.04, 1.43) | 338 | 1.14  (0.97, 1.34) | | 0.085 | | |  | | |
|  |  |  | Ref |  | 1.03  (0.87, 1.22) |  | 1.18  (0.99, 1.40) |  | 1.10  (0.92, 1.31) | | 0.327 | | |  | | |
| 4-Nitrophenol | 263 | | Ref | 318 | 1.17  (0.99, 1.38) | 326 | 1.19  (1.01, 1.40)* | 349 | 1.25  (1.06, 1.47) | | 0.021* | | |  | | |
|  |  |  | Ref |  | 1.12  (0.95, 1.33) |  | 1.11  (0.93, 1.31) |  | 1.20  (1.00, 1.43)* | | 0.084 | | |  | | |
| Selenium Compounds | 291 | | Ref | 303 | 1.04  (0.88, 1.22) | 356 | 1.17  (1.00, 1.37)* | 306 | 1.01  (0.86, 1.18) | | 0.915 | | |  | | |
|  |  |  | Ref |  | 1.03  (0.87, 1.21) |  | 1.09  (0.93, 1.29) |  | 0.91  (0.76, 1.09) | | 0.179 | | |  | | |
| Styrene^a^ | 387 | | Ref | 258 | 1.04  (0.88, 1.21) | 295 | 0.99  (0.85, 1.15) | 316 | 0.92  (0.80, 1.07) | | 0.812 | | |  | | |
|  |  |  | Ref |  | 1.00  (0.85, 1.18) |  | 0.96  (0.82, 1.12) |  | 0.93  (0.80, 1.08) | | 0.301 | | |  | | |
| **^a^** Basic models are adjusted for time-varying current age and calendar period by stratification.  **^b^** All fully adjusted models are adjusted for time-varying current age and calendar period by stratification and include terms for race, family history of breast cancer, history of aspiration or biopsy confirmed benign breast disease, age at menarche, parity and age at first birth, menopausal status and postmenopausal hormone use, oral contraception use, recent mammogram, height, BMI at age 18, difference between current BMI and BMI at age 18, physical activity, overall diet quality (including alcohol consumption), alcohol consumption at age 15 and age 18, current smoking status, shift work, individual-level SES (marital status, living arrangements, household income), area-level SES (Census tract median home value and median income), and Census region of residence  ^a^ Diesel exhaust and Styrene are both potential estrogen disruptors and mammary carcinogens  Grey boxes indicate HAPs without enough variability to create quartiles; ***** indicates p-values<0.05 | | | | | | | | | | | |  | | |  |  |

| **Supplemental Table S5: Correlations between exposures based on the 1996 and 2002 NATA values for HAPs available in both time periods among women in the Nurses’ Health Study II cohort** | | | | |
| --- | --- | --- | --- | --- |
| **Hazardous Air Pollutant** | **Pearson Correlation** | **p-value** | **Spearman Correlation** | **p-value** |
| 1,3-Butadiene | 0.59 | <0.0001 | 0.74 | <0.0001 |
| Acrylonitrile | 0.11 | <0.0001 | 0.49 | <0.0001 |
| Benzene (Including Benzene From Gasoline) | 0.72 | <0.0001 | 0.79 | <0.0001 |
| Carbon Tetrachloride | 0.07 | <0.0001 | 0.10 | <0.0001 |
| Diesel Engine Emissions | 0.47 | <0.0001 | 0.85 | <0.0001 |
| Ethylene Dibromide (Dibromoethane) | 0.12 | <0.0001 | -0.02 | <0.0001 |
| Ethylene Dichloride (1,2-Dichloroethane) | 0.10 | <0.0001 | 0.35 | <0.0001 |
| Ethylene Oxide | 0.53 | <0.0001 | 0.42 | <0.0001 |
| Hydrazine | 0.14 | <0.0001 | 0.27 | <0.0001 |
| Methylene Chloride (Dichloromethane) | 0.53 | <0.0001 | 0.78 | <0.0001 |
| Propylene Dichloride (1,2-Dichloropropane) | 0.09 | <0.0001 | 0.37 | <0.0001 |
| Vinyl Chloride | 0.12 | <0.0001 | 0.67 | <0.0001 |

| **Supplemental Table S6: Multivariable adjusted associations of increasing quartiles of each HAP exposure based on the 1996 NATA estimates on risk of incident invasive overall, estrogen-receptor positive (ER+) or estrogen-receptor negative (ER-) breast cancer 1989-2011 among 109,239 members of the Nurses’ Health Study II cohort.** | | | | | | | | | | |
| --- | --- | --- | --- | --- | --- | --- | --- | --- | --- | --- |
| **Hazardous Air Pollutant** |  | **Quartile 1** | | **Quartile 2** | | **Quartile 3** | | **Quartile 4** | |  |
|  | **Outcome** | **Cases** | **HR (95% CI)** | **Cases** |  | **Cases** |  | **Cases** |  | **p-value for trend** |
|  |  |  |  |  | **HR**  **(95% CI)** |  | **HR**  **(95% CI)** |  | **HR**  **(95% CI)** |  |
| 1,3-Butadiene | Overall | 658 | Ref | 730 | 1.07  (0.96, 1.19) | 665 | 0.97  (0.87, 1.08) | 714 | 1.10  (0.98, 1.23) | 0.26 |
|  | ER+ | 404 | Ref | 496 | 1.19  (1.04, 1.36)* | 417 | 1.01  (0.87, 1.16) | 470 | 1.20  (1.04, 1.38)* | 0.11 |
|  | ER- | 124 | Ref | 114 | 0.92  (0.70, 1.19) | 117 | 0.93  (0.72, 1.21) | 118 | 1.00  (0.76, 1.30) | 0.88 |
| Acrylonitrile | Overall | 676 | Ref | 721 | 1.01  (0.91, 1.13) | 691 | 0.97  (0.87, 1.08) | 679 | 0.96  (0.87, 1.07) | 0.21 |
|  | ER+ | 429 | Ref | 484 | 1.06  (0.93, 1.22) | 439 | 0.97  (0.84, 1.12) | 435 | 0.97  (0.84, 1.12) | 0.33 |
|  | ER- | 119 | Ref | 117 | 0.95  (0.73, 1.24) | 119 | 0.98  (0.75, 1.28) | 118 | 0.98  (0.75, 1.29) | 0.98 |
| Benzene (Including Benzene From Gasoline) | Overall | 648 | Ref | 742 | 1.10  (0.99, 1.22) | 666 | 0.98  (0.88, 1.10) | 711 | 1.07  (0.95, 1.20) | 0.77 |
|  | ER+ | 402 | Ref | 496 | 1.20  (1.05, 1.37)* | 424 | 1.03  (0.89, 1.19) | 465 | 1.15  (1.00, 1.33) | 0.36 |
|  | ER- | 120 | Ref | 118 | 0.98  (0.75, 1.27) | 119 | 0.98  (0.75, 1.29) | 116 | 1.01  (0.76, 1.33) | 0.99 |
| Carbon Tetrachloride | Overall | 670 | Ref | 682 | 0.97  (0.87, 1.09) | 698 | 0.97  (0.87, 1.08) | 717 | 1.00  (0.89, 1.13) | 0.99 |
|  | ER+ | 432 | Ref | 451 | 1.00  (0.87, 1.14) | 446 | 0.97  (0.84, 1.12) | 458 | 1.00  (0.86, 1.16) | 0.87 |
|  | ER- | 122 | Ref | 115 | 0.91  (0.70, 1.19) | 129 | 1.01  (0.77, 1.31) | 107 | 0.89  (0.67, 1.19) | 0.45 |
| Diesel Engine Emissions | Overall | 664 | Ref | 677 | 1.00  (0.90, 1.12) | 674 | 0.98  (0.87, 1.09) | 752 | 1.10  (0.98, 1.24) | 0.18 |
|  | ER+ | 432 | Ref | 437 | 1.00  (0.88, 1.15) | 431 | 0.97  (0.84, 1.11) | 487 | 1.10  (0.96, 1.27) | 0.25 |
|  | ER- | 117 | Ref | 114 | 0.95  (0.73, 1.24) | 117 | 0.97  (0.74, 1.27) | 125 | 1.14  (0.86, 1.50) | 0.34 |
| Ethylene Dibromide (Dibromoethane) | Overall | 696 | Ref | 697 | 0.96  (0.86, 1.06) | 658 | 0.90  (0.81, 1.01) | 716 | 0.98  (0.88, 1.10) | 0.82 |
|  | ER+ | 447 | Ref | 462 | 0.99  (0.87, 1.13) | 416 | 0.89  (0.78, 1.03) | 462 | 1.00  (0.86, 1.15) | 0.78 |
|  | ER- | 123 | Ref | 114 | 0.89  (0.68, 1.16) | 112 | 0.91  (0.70, 1.19) | 124 | 1.02  (0.78, 1.33) | 0.43 |
| Ethylene Dichloride (1,2-Dichloroethane) | Overall | 685 | Ref | 730 | 1.03  (0.92, 1.15) | 663 | 0.92  (0.82, 1.03) | 689 | 0.95  (0.85, 1.07) | 0.26 |
|  | ER+ | 437 | Ref | 495 | 1.09  (0.96, 1.25) | 418 | 0.91  (0.79, 1.05) | 437 | 0.95  (0.82, 1.10) | 0.19 |
|  | ER- | 117 | Ref | 115 | 0.97  (0.75, 1.26) | 117 | 1.00  (0.76, 1.30) | 124 | 1.11  (0.84, 1.46) | 0.35 |
| Ethylene Oxide | Overall | 664 | Ref | 733 | 1.06  (0.95, 1.18) | 682 | 0.99  (0.89, 1.11) | 688 | 1.00  (0.89, 1.12) | 0.54 |
|  | ER+ | 411 | Ref | 476 | 1.13  (0.99, 1.29) | 453 | 1.09  (0.95, 1.25) | 447 | 1.07  (0.93, 1.24) | 0.95 |
|  | ER- | 125 | Ref | 123 | 0.96  (0.74, 1.24) | 106 | 0.84  (0.64, 1.10) | 119 | 0.95  (0.73, 1.25) | 0.82 |
| Hydrazine | Overall | 685 | Ref | 716 | 1.00  (0.90, 1.11) | 691 | 0.98  (0.88, 1.10) | 675 | 0.98  (0.87, 1.09) | 0.54 |
|  | ER+ | 436 | Ref | 478 | 1.06  (0.93, 1.21) | 449 | 1.01  (0.88, 1.16) | 424 | 0.97  (0.84, 1.12) | 0.26 |
|  | ER- | 127 | Ref | 103 | 0.78  (0.60, 1.02) | 126 | 1.00  (0.77, 1.29) | 117 | 0.98  (0.75, 1.28) | 0.55 |
| Methylene Chloride (Dichloromethane) | Overall | 694 | Ref | 707 | 0.97  (0.87, 1.08) | 668 | 0.93  (0.83, 1.04) | 698 | 1.00  (0.89, 1.12) | 0.88 |
|  | ER+ | 448 | Ref | 466 | 1.01  (0.88, 1.15) | 420 | 0.92  (0.80, 1.05) | 453 | 1.00  (0.87, 1.15) | 0.80 |
|  | ER- | 122 | Ref | 109 | 0.88  (0.67, 1.14) | 128 | 1.05  (0.81, 1.36) | 114 | 1.01  (0.77, 1.33) | 0.64 |
| Propylene Dichloride (1,2-Dichloropropane) | Overall | 674 | Ref | 730 | 1.03  (0.93, 1.15) | 685 | 0.96  (0.85, 1.07) | 678 | 0.95  (0.84, 1.07) | 0.13 |
|  | ER+ | 425 | Ref | 479 | 1.07  (0.94, 1.22) | 445 | 0.98  (0.85, 1.12) | 438 | 0.97  (0.84, 1.13) | 0.30 |
|  | ER- | 119 | Ref | 114 | 0.94  (0.72, 1.22) | 118 | 0.97  (0.74, 1.27) | 122 | 1.04  (0.78, 137) | 0.62 |
| Vinyl Chloride | Overall | 663 | Ref | 733 | 1.06  (0.95, 1.18) | 685 | 0.97  (0.87, 1.09) | 686 | 0.96  (0.86, 1.09) | 0.17 |
|  | ER+ | 413 | Ref | 496 | 1.15  (1.01, 1.32)* | 436 | 0.99  (0.86, 1.14) | 442 | 1.00  (0.86, 1.16) | 0.27 |
|  | ER- | 119 | Ref | 112 | 0.91  (0.70, 1.19) | 121 | 1.00  (0.76, 1.30) | 121 | 1.05  (0.79, 1.39) | 0.55 |
| \| **Note:** All models adjusted for age, calendar period, race, family history of breast cancer, history of aspiration or biopsy confirmed benign breast disease, age at menarche, parity and age at first birth, menopausal status and postmenopausal hormone use, oral contraception use, recent mammogram, height, BMI at age 18, difference between current BMI and BMI at age 18, smoking status, physical activity, overall diet quality (including alcohol consumption), alcohol consumption at age 15 and age 18, rotating shift work, individual-level SES (marital status, living arrangements, household income), area-level SES (Census tract median home value and median income), and Census region of residence \| **Note:** All models adjusted for age, calendar period, race, family history of breast cancer, history of aspiration or biopsy confirmed benign breast disease, age at menarche, parity and age at first birth, menopausal status and postmenopausal hormone use, oral contraception use, recent mammogram, height, BMI at age 18, difference between current BMI and BMI at age 18, smoking status, physical activity, overall diet quality, alcohol consumption at age 15 and age 18, individual-level SES (marital status, living arrangements, household income), area-level SES (Census tract median home value and median income), and Census region of residence \| \| --- \| --- \| \| Grey boxes indicate HAPs without enough variability to create quartiles; ***** indicates p-values<0.05 \| * Styrene is both a potential estrogen disruptor and a mammary carcinogen \| | | | | | | | | | | |

| **Supplemental Figure S1: Associations of increasing quartiles of diesel exhaust (top panel) or styrene (bottom panel) exposure on risk of incident invasive breast cancer 1989-2011 among women in the Nurses’ Health Study II cohort. Hazard ratios (HR) and 95 % confidence intervals (95%CI) are presented for basic models, for models adding each potential confounder (or group of confounders) to the basic model, and for fully adjusted models.** |
| --- |
| 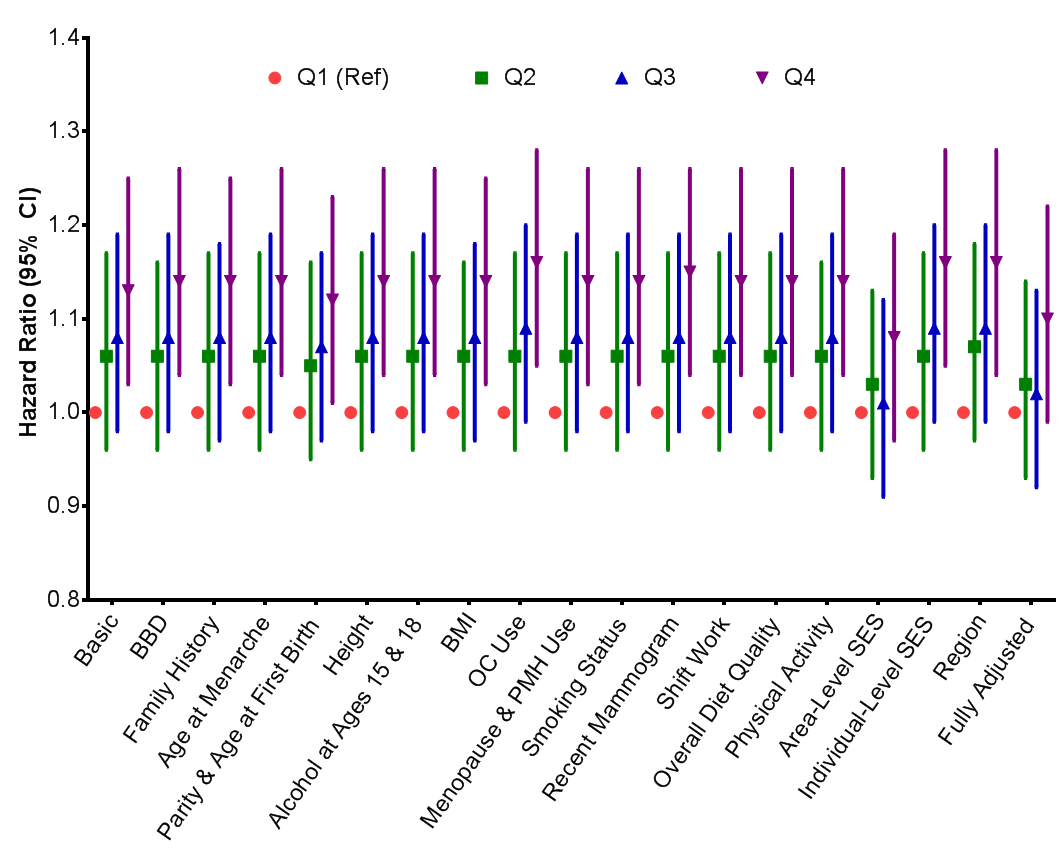 |
| 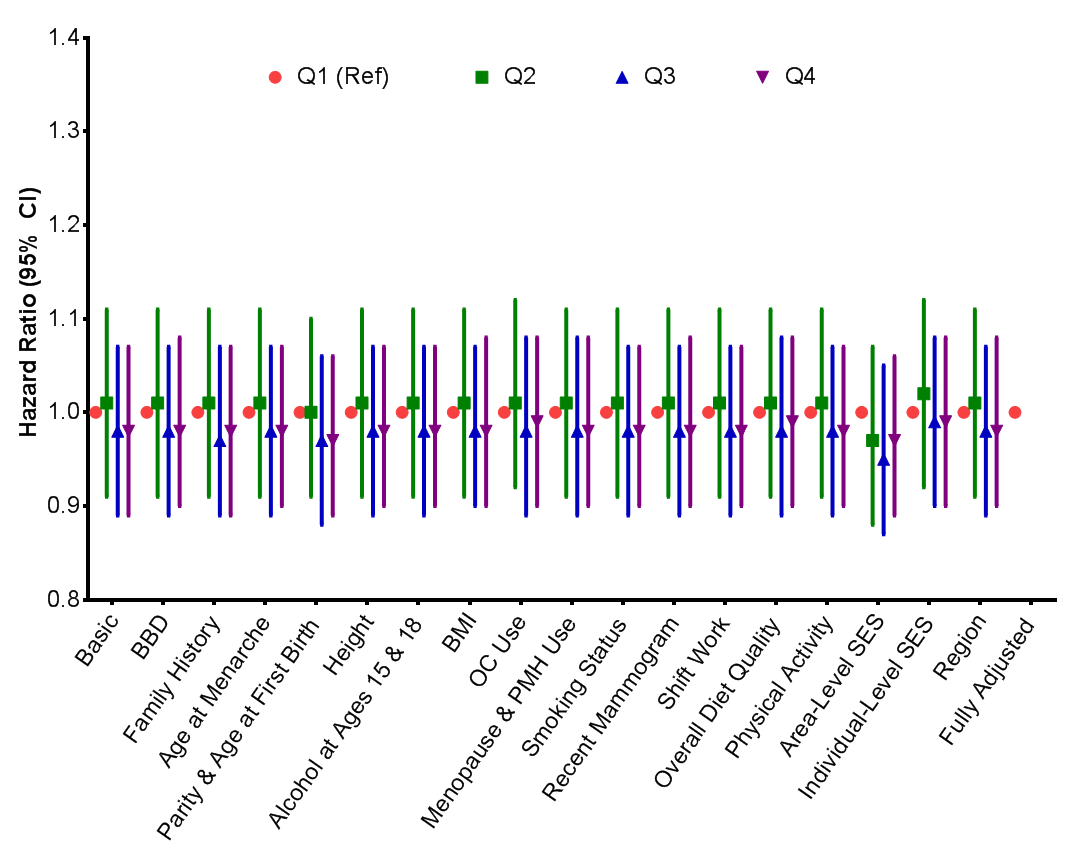 |
