# Supplementary material for: Exposure to hazardous air pollutants and risk of incident breast cancer in the nurses’ health study II
Source: Environ Health. 2018 Mar 27;17:28. doi: 10.1186/s12940-018-0372-3 (PMC5870204; doi:10.1186/s12940-018-0372-3)
Supplement: Supplementary file 2 — Multivariable adjusted associations of increasing quartiles of each mammary carcinogen HAP exposure on risk of incident invasive overall, estrogen-receptor positive (ER+) or estrogen-receptor negative (ER-) breast cancer 1989–2011 among 109,239 members of the Nurses’ Health Study II cohort (DOCX 51 kb) [file 12940_2018_372_MOESM2_ESM.docx]

Additional file 2: Multivariable adjusted associations of increasing quartiles of each mammary carcinogen HAP exposure on risk of incident invasive overall, estrogen-receptor positive (ER+) or estrogen-receptor negative (ER-) breast cancer 1989–2011 among 109,239 members of the Nurses’ Health Study II cohort

| Hazardous Air Pollutant |  | Quartile 1 | | Quartile 2 | | Quartile 3 | | Quartile 4 | |  |
| --- | --- | --- | --- | --- | --- | --- | --- | --- | --- | --- |
|  | Outcome | Cases | HR (95% CI) | Cases | HR (95% CI) | Cases | HR (95% CI) | Cases | HR (95% CI) | *p*-value for trend |
| 1,2-Dibromo-3-Chloropropane | Overall | 957 | Ref | 692 | 0.97 (0.86, 1.09) | 757 | 0.96 (0.84, 1.10) | 915 | 1.12 (0.98, 1.29) | 0.004***** |
|  | ER+ | 639 | Ref | 447 | 0.94 (0.81, 1.09) | 491 | 0.94 (0.79, 1.11) | 554 | 1.09 (0.92, 1.30) | 0.028* |
|  | ER- | 153 | Ref | 135 | 1.16 (0.87, 1.55) | 131 | 1.07 (0.77, 1.50) | 139 | 1.14 (0.81, 1.61) | 0.728 |
| 1,3-Butadiene | Overall | 706 | Ref | 993 | 0.97 (0.87, 1.07) | 776 | 1.04 (0.93, 1.16) | 846 | 0.98 (0.87, 1.09) | 0.892 |
|  | ER+ | 445 | Ref | 643 | 1.00 (0.88, 1.13) | 518 | 1.11 (0.97, 1.27) | 554 | 1.01 (0.88, 1.17) | 0.697 |
|  | ER- | 122 | Ref | 179 | 1.06 (0.83, 1.35) | 133 | 1.09 (0.84, 1.42) | 124 | 0.90 (0.68, 1.18) | 0.325 |
| 1,4-Dioxane | Overall | 1807 | Ref | 688 | 0.93 (0.85, 1.02) | 826 | 0.93 (0.84, 1.03) |  |  | 0.351 |
|  | ER+ | 1186 | Ref | 437 | 0.90 (0.81, 1.01) | 537 | 0.93 (0.82, 1.05) |  |  | 0.471 |
|  | ER- | 307 | Ref | 125 | 1.05 (0.84, 1.30) | 126 | 0.90 (0.70, 1.15) |  |  | 0.301 |
| 2,4-Dinitrotoluene | Overall | 827 | Ref | 839 | 1.07 (0.94, 1.21) | 875 | 1.10 (0.97, 1.25) | 789 | 1.01 (0.88, 1.15) | 0.387 |
|  | ER+ | 539 | Ref | 549 | 1.16 (0.99, 1.37) | 567 | 1.19 (1.01, 1.39)* | 505 | 1.07 (0.90, 1.26) | 0.516 |
|  | ER- | 138 | Ref | 143 | 1.00 (0.74, 1.34) | 148 | 1.02 (0.75, 1.39) | 129 | 0.96 (0.70, 1.31) | 0.705 |
| 2,4-Toluene Diisocyanate | Overall | 812 | Ref | 797 | 0.95 (0.86, 1.05) | 890 | 1.07 (0.97, 1.18) | 822 | 1.02 (0.92, 1.13) | 0.498 |
|  | ER+ | 514 | Ref | 548 | 1.04 (0.91, 1.17) | 571 | 1.09 (0.96, 1.23) | 527 | 1.05 (0.92, 1.20) | 0.626 |
|  | ER- | 149 | Ref | 133 | 0.88 (0.69, 1.12) | 147 | 1.00 (0.79, 1.28) | 129 | 0.97 (0.74, 1.25) | 0.921 |
| 2-Chloroacetophenone | Overall | 1032 | Ref | 719 | 0.97 (0.87, 1.09) | 825 | 0.92 (0.82, 1.03) | 745 | 0.84 (0.75, 0.94)* | 0.001* |
|  | ER+ | 703 | Ref | 463 | 0.91 (0.79, 1.04) | 529 | 0.85 (0.74, 0.98)* | 465 | 0.77 (0.67, 0.88)* | 0.005* |
|  | ER- | 159 | Ref | 117 | 1.02 (0.76, 1.35) | 143 | 1.05 (0.80, 1.38) | 139 | 1.01 (0.76, 1.32) | 0.932 |
| Acrylonitrile | Overall | 850 | Ref | 825 | 0.97 (0.88, 1.07) | 805 | 0.97 (0.88, 1.08) | 841 | 0.99 (0.89, 1.11) | 0.876 |
|  | ER+ | 559 | Ref | 534 | 0.95 (0.85, 1.08) | 520 | 0.94 (0.82, 1.07) | 547 | 0.96 (0.83, 1.10) | 0.505 |
|  | ER- | 147 | Ref | 130 | 0.87 (0.68, 1.11) | 153 | 1.02 (0.79, 1.31) | 128 | 0.86 (0.65, 1.13) | 0.546 |
| Benzene (Including Benzene From Gasoline) | Overall | 799 | Ref | 861 | 1.04 (0.94, 1.14) | 835 | 0.99 (0.89, 1.09) | 826 | 1.03 (0.93, 1.14) | 0.756 |
|  | ER+ | 501 | Ref | 565 | 1.09 (0.96, 1.24) | 559 | 1.07 (0.94, 1.21) | 535 | 1.08 (0.95, 1.23) | 0.356 |
|  | ER- | 158 | Ref | 135 | 0.84 (0.66, 1.07) | 132 | 0.81 (0.63, 1.04) | 133 | 0.86 (0.67, 1.10) | 0.263 |
| Benzidine | Overall | 1464 | Ref | 175 | 0.94 (0.80, 1.10) | 866 | 0.99 (0.89, 1.10) | 816 | 0.97 (0.87, 1.08) | 0.597 |
|  | ER+ | 950 | Ref | 114 | 0.94 (0.77, 1.15) | 585 | 1.04 (0.91, 1.18) | 511 | 0.96 (0.84, 1.10) | 0.412 |
|  | ER- | 270 | Ref | 27 | 0.79 (0.53, 1.17) | 123 | 0.77 (0.59, 1.00) | 138 | 0.89 (0.69, 1.16) | 0.921 |
| Carbon Tetrachloride | Overall | 3303 | Ref | 18 | 1.02 (0.64, 1.63) |  |  |  |  |  |
|  | ER+ | 2148 | Ref | 12 | 1.06 (0.60, 1.87) |  |  |  |  |  |
|  | ER- | 556 | Ref | 2 | 0.75 (0.19, 3.02) |  |  |  |  |  |
| Chloroprene | Overall | 857 | Ref | 820 | 0.98 (0.87, 1.09) | 898 | 1.04 (0.93, 1.16) | 746 | 0.89 (0.80, 0.99)* | 0.015* |
|  | ER+ | 559 | Ref | 537 | 1.02 (0.89, 1.16) | 582 | 1.08 (0.94, 1.23) | 482 | 0.92 (0.80, 1.05) | 0.076 |
|  | ER- | 149 | Ref | 141 | 0.90 (0.69, 1.17) | 155 | 0.98 (0.76, 1.27) | 113 | 0.78 (0.59, 1.03) | 0.080 |
| Diesel Engine Emissions^a^ | Overall | 795 | Ref | 828 | 1.03 (0.93, 1.14) | 828 | 1.02 (0.92, 1.13) | 870 | 1.10 (0.99, 1.22) | 0.096 |
|  | ER+ | 518 | Ref | 530 | 1.02 (0.90, 1.15) | 540 | 1.03 (0.91, 1.17) | 572 | 1.11 (0.97, 1.27) | 0.099 |
|  | ER- | 139 | Ref | 152 | 1.07 (0.84, 1.35) | 146 | 1.02 (0.80, 1.32) | 121 | 0.91 (0.70, 1.19) | 0.401 |
| Ethylene Dibromide (Dibromoethane) | Overall | 920 | Ref | 785 | 0.89 (0.81, 0.99)* | 812 | 0.93 (0.83, 1.05) | 804 | 0.91 (0.80, 1.02) | 0.378 |
|  | ER+ | 610 | Ref | 515 | 0.91 (0.80, 1.03) | 524 | 0.94 (0.82, 1.09) | 511 | 0.89 (0.77, 1.04) | 0.283 |
|  | ER- | 144 | Ref | 129 | 0.90 (0.70, 1.16) | 154 | 1.02 (0.77, 1.35) | 131 | 0.83 (0.62, 1.12) | 0.194 |
| Ethylene Dichloride (1,2-Dichloroethane) | Overall | 842 | Ref | 804 | 0.96 (0.87, 1.06) | 833 | 1.00 (0.90, 1.10) | 842 | 1.01 (0.91, 1.11) | 0.647 |
|  | ER+ | 557 | Ref | 527 | 0.96 (0.85, 1.08) | 534 | 0.98 (0.87, 1.11) | 542 | 1.00 (0.88, 1.13) | 0.824 |
|  | ER- | 143 | Ref | 141 | 1.00 (0.79, 1.27) | 145 | 1.05 (0.83, 1.33) | 129 | 0.99 (0.77, 1.28) | 0.944 |
| Ethylene Oxide | Overall | 833 | Ref | 791 | 0.98 (0.89, 1.09) | 869 | 1.07 (0.97, 1.18) | 828 | 1.04 (0.94, 1.15) | 0.248 |
|  | ER+ | 560 | Ref | 517 | 0.98 (0.86, 1.10) | 565 | 1.05 (0.93, 1.19) | 518 | 0.99 (0.87, 1.12) | 0.931 |
|  | ER- | 137 | Ref | 130 | 0.96 (0.75, 1.23) | 157 | 1.17 (0.92, 1.48) | 134 | 1.04 (0.81, 1.33) | 0.472 |
| Ethylidene Dichloride (1,1-Dichloroethane) | Overall | 808 | Ref | 838 | 1.03 (0.93, 1.14) | 811 | 1.00 (0.90, 1.10) | 864 | 1.04 (0.92, 1.16) | 0.638 |
|  | ER+ | 521 | Ref | 552 | 1.05 (0.93, 1.19) | 522 | 0.99 (0.88, 1.13) | 565 | 1.04 (0.90, 1.21) | 0.722 |
|  | ER- | 137 | Ref | 146 | 1.06 (0.84, 1.34) | 142 | 1.04 (0.82, 1.32) | 133 | 0.99 (0.74, 1.32) | 0.718 |
| Hydrazine | Overall | 1021 | Ref | 628 | 1.02 (0.92, 1.13) | 930 | 1.12 (1.02, 1.23)* | 742 | 0.92 (0.83, 1.01) | 0.005* |
|  | ER+ | 695 | Ref | 415 | 0.99 (0.87, 1.12) | 592 | 1.05 (0.93, 1.17) | 458 | 0.84 (0.75, 0.95)* | 0.011* |
|  | ER- | 163 | Ref | 111 | 1.08 (0.84, 1.38) | 160 | 1.24 (0.99, 1.55) | 124 | 1.01 (0.79, 1.28) | 0.495 |
| Methylene Chloride (Dichloromethane) | Overall | 779 | Ref | 849 | 0.95 (0.86, 1.05) | 849 | 1.00 (0.91, 1.11) | 844 | 1.02 (0.91, 1.14) | 0.527 |
|  | ER+ | 508 | Ref | 565 | 0.97 (0.86, 1.10) | 531 | 0.96 (0.84, 1.09) | 556 | 1.00 (0.87, 1.16) | 0.855 |
|  | ER- | 131 | Ref | 143 | 1.00 (0.78, 1.28) | 153 | 1.15 (0.90, 1.48) | 131 | 1.04 (0.79, 1.38) | 0.699 |
| Nitrobenzene | Overall | 850 | Ref | 792 | 0.95 (0.85, 1.05) | 875 | 1.03 (0.92, 1.14) | 804 | 0.96 (0.86, 1.08) | 0.659 |
|  | ER+ | 547 | Ref | 523 | 1.02 (0.89, 1.16) | 573 | 1.10 (0.96, 1.26) | 517 | 1.02 (0.89, 1.17) | 0.899 |
|  | ER- | 150 | Ref | 137 | 0.85 (0.66, 1.11) | 149 | 0.92 (0.71, 1.19) | 122 | 0.83 (0.63, 1.09) | 0.334 |
| O-Toluidine | Overall | 884 | Ref | 797 | 0.89 (0.79, 0.99)* | 866 | 0.96 (0.86, 1.07) | 774 | 0.89 (0.80, 0.99)* | 0.190 |
|  | ER+ | 566 | Ref | 528 | 0.96 (0.84, 1.10) | 581 | 1.06 (0.93, 1.21) | 485 | 0.93 (0.81, 1.06) | 0.172 |
|  | ER- | 153 | Ref | 131 | 0.79 (0.61, 1.02) | 150 | 0.92 (0.71, 1.18) | 124 | 0.83 (0.63, 1.08) | 0.505 |
| Propylene Dichloride (1,2-Dichloropropane) | Overall | 830 | Ref | 818 | 0.94 (0.85, 1.04) | 807 | 0.95 (0.85, 1.06) | 866 | 1.00 (0.87, 1.15) | 0.472 |
|  | ER+ | 559 | Ref | 526 | 0.88 (0.78, 1.00) | 525 | 0.88 (0.77, 1.01) | 550 | 0.89 (0.74, 1.05) | 0.659 |
|  | ER- | 143 | Ref | 138 | 0.96 (0.75, 1.23) | 127 | 0.96 (0.74, 1.25) | 150 | 1.25 (0.89, 1.74) | 0.074 |
| Propylene Oxide | Overall | 833 | Ref | 830 | 1.01 (0.91, 1.12) | 827 | 1.00 (0.90, 1.12) | 831 | 1.03 (0.93, 1.15) | 0.582 |
|  | ER+ | 543 | Ref | 549 | 1.07 (0.94, 1.22) | 523 | 1.01 (0.88, 1.15) | 545 | 1.08 (0.94, 1.23) | 0.407 |
|  | ER- | 135 | Ref | 143 | 1.04 (0.81, 1.34) | 154 | 1.12 (0.87, 1.45) | 126 | 0.96 (0.73, 1.25) | 0.486 |
| Styrene^a^ | Overall | 1050 | Ref | 632 | 0.97 (0.88, 1.07) | 753 | 0.94 (0.85, 1.03) | 886 | 0.97 (0.89, 1.06) | 0.676 |
|  | ER+ | 681 | Ref | 400 | 0.96 (0.85, 1.09) | 495 | 0.97 (0.86, 1.09) | 584 | 1.01 (0.90, 1.13) | 0.744 |
|  | ER- | 176 | Ref | 113 | 1.05 (0.83, 1.34) | 131 | 0.99 (0.78, 1.24) | 138 | 0.92 (0.73, 1.15) | 0.341 |
| Vinyl Chloride | Overall | 795 | Ref | 809 | 0.99 (0.89, 1.09) | 899 | 1.08 (0.98, 1.20) | 818 | 0.98 (0.88, 1.10) | 0.526 |
|  | ER+ | 533 | Ref | 523 | 0.93 (0.82, 1.06) | 583 | 1.03 (0.91, 1.17) | 521 | 0.90 (0.78, 1.04) | 0.146 |
|  | ER- | 127 | Ref | 154 | 1.17 (0.92, 1.49) | 157 | 1.21 (0.94, 1.55) | 120 | 0.96 (0.72, 1.29) | 0.288 |
| Vinylidene Chloride (1,1-Dichloroethylene) | Overall | 819 | Ref | 860 | 1.03 (0.93, 1.13) | 855 | 1.02 (0.93, 1.13) | 787 | 0.96 (0.87, 1.06) | 0.230 |
|  | ER+ | 519 | Ref | 574 | 1.10 (0.97, 1.24) | 554 | 1.06 (0.94, 1.20) | 513 | 1.01 (0.89, 1.15) | 0.621 |
|  | ER- | 152 | Ref | 126 | 0.81 (0.64, 1.04) | 166 | 1.09 (0.87, 1.38) | 114 | 0.79 (0.61, 1.02) | 0.165 |

Note: All models adjusted for age, calendar period, race, family history of breast cancer, history of aspiration or biopsy confirmed benign breast disease, age at menarche, parity and age at first birth, menopausal status and postmenopausal hormone use, oral contraception use, recent mammogram, height, BMI at age 18, difference between current BMI and BMI at age 18, smoking status, physical activity, overall diet quality (including alcohol consumption), alcohol consumption at age 15 and age 18, individual-level SES (marital status, living arrangements, household income), shift work, area-level SES (Census tract median home value and median income), and Census region of residence

^a^Diesel exhaust and Styrene are both potential estrogen disruptors and mammary carcinogens

Grey boxes indicate HAPs without enough variability to create quartiles; *indicates *p*-values<0.05
